# Supplementary material for: Maternal pluripotency factors initiate extensive chromatin remodelling to predefine first response to inductive signals
Source: Nat Commun. 2019 Sep 19;10:4269. doi: 10.1038/s41467-019-12263-w (PMC6753111; doi:10.1038/s41467-019-12263-w)
Supplement: Supplementary file 1 — Supplementary Information [file 41467_2019_12263_MOESM1_ESM.pdf]

Maternal pluripotency factors initiate extensive chromatin remodelling to predefine first response to inductive signals

Gentsch et al.

Supplementary Figures

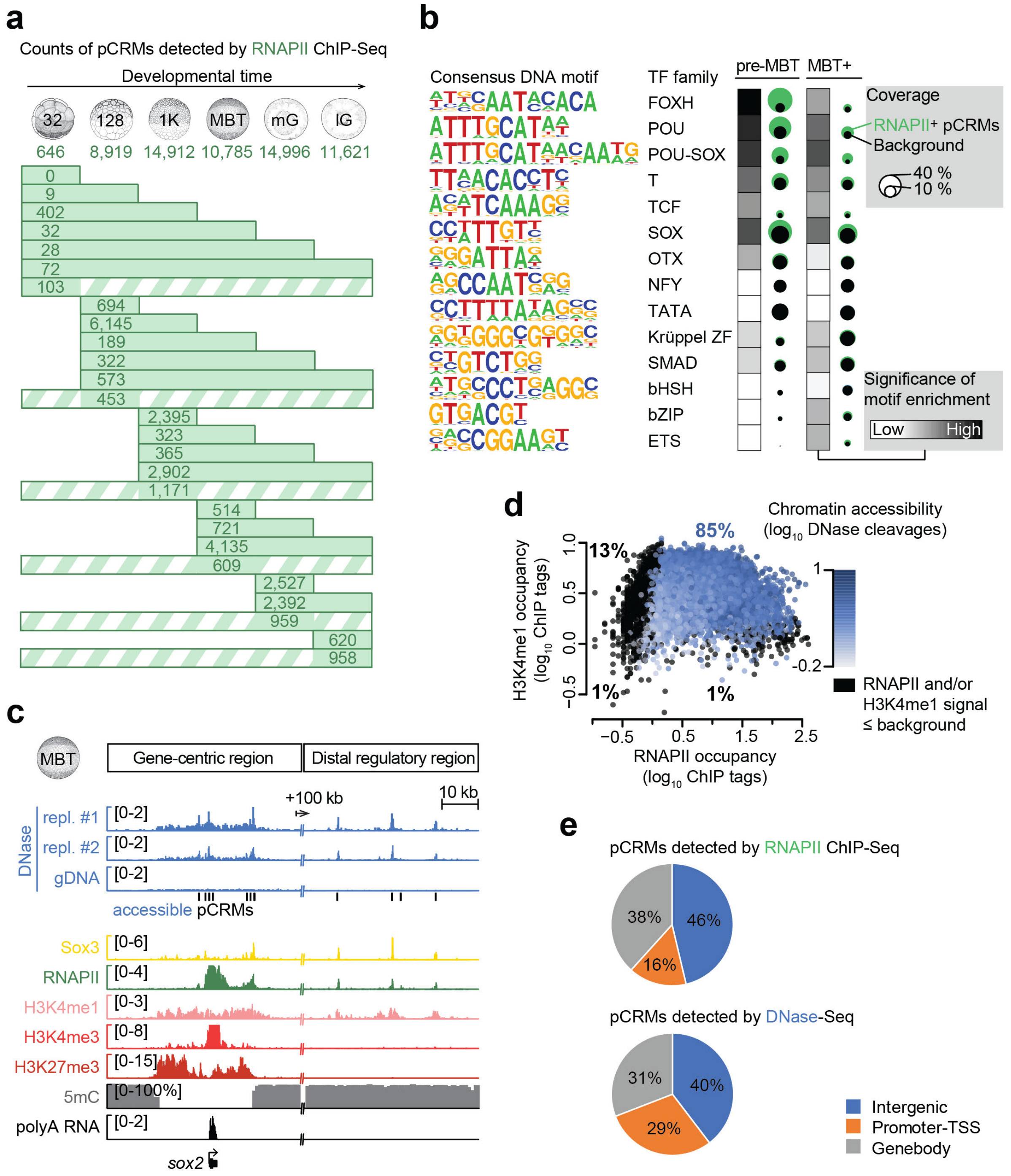

Supplementary Fig. 1 Characterisation of pCRMs instructing ZGA. (**a**) Graph shows the temporal dynamics of RNAPII-engaged (RNAPII^{+}) pCRMs (≥1 ChIP tag per million) from the 32-cell to the late gastrula stage. Striped bars refer to RNAPII^{+} pCRMs fragmentarily detected across consecutive developmental stages. Abbreviations used for the developmental timeline: 32, 128 and 1K, 32-, 128- and 1,024-cell stage; MBT, mid-blastula transition; mG and lG, mid- and late gastrula stage. (**b**) Heat map and bubble plot show the respective statistical significance (hypergeometric p-value) and coverage of DNA motifs enriched among the top 5,000 RNAPII^{+} pCRMs detected from the 32-cell to the 1,024-cell stage (pre-MBT) and from the MBT to the late gastrula stage (MBT+). This analysis included 39,785 (pre-MBT) and 41,898 (MBT+) genomic 'background' regions matching the overall GC contents of the selected RNAPII^{+} pCRMs. (**c**) Validation of our DNase-Seq method: DNase-probed chromatin accessibility (biological replicates #1 and #2) at the *sox2* locus (gene-centric region and downstream distal regulatory region) is shown alongside with various other chromatin features (Sox3, RNAPII, H3K4me1, poly(A) RNA from

this study and H3K4me3, H3K27me3, 5-methylcytosine [5mC] from ref. 1) detected around the MBT. DNase-treated naked genomic DNA (gDNA) was used as a negative control for DNase-probed chromatin accessibility. (**d**) Biplot shows the DNA occupancy levels of RNAPII and H3K4me1 at accessible pCRMs. pCRMs (dots) are colored according to their chromatin accessibility except for the pCRMs that showed RNAPII and/or H3K4me1 signals piled up over a distance of 1 kb below background. (**e**) Pie charts show the genomic distribution of pCRMs found by RNAPII enrichment or DNase hypersensitivity, respectively.

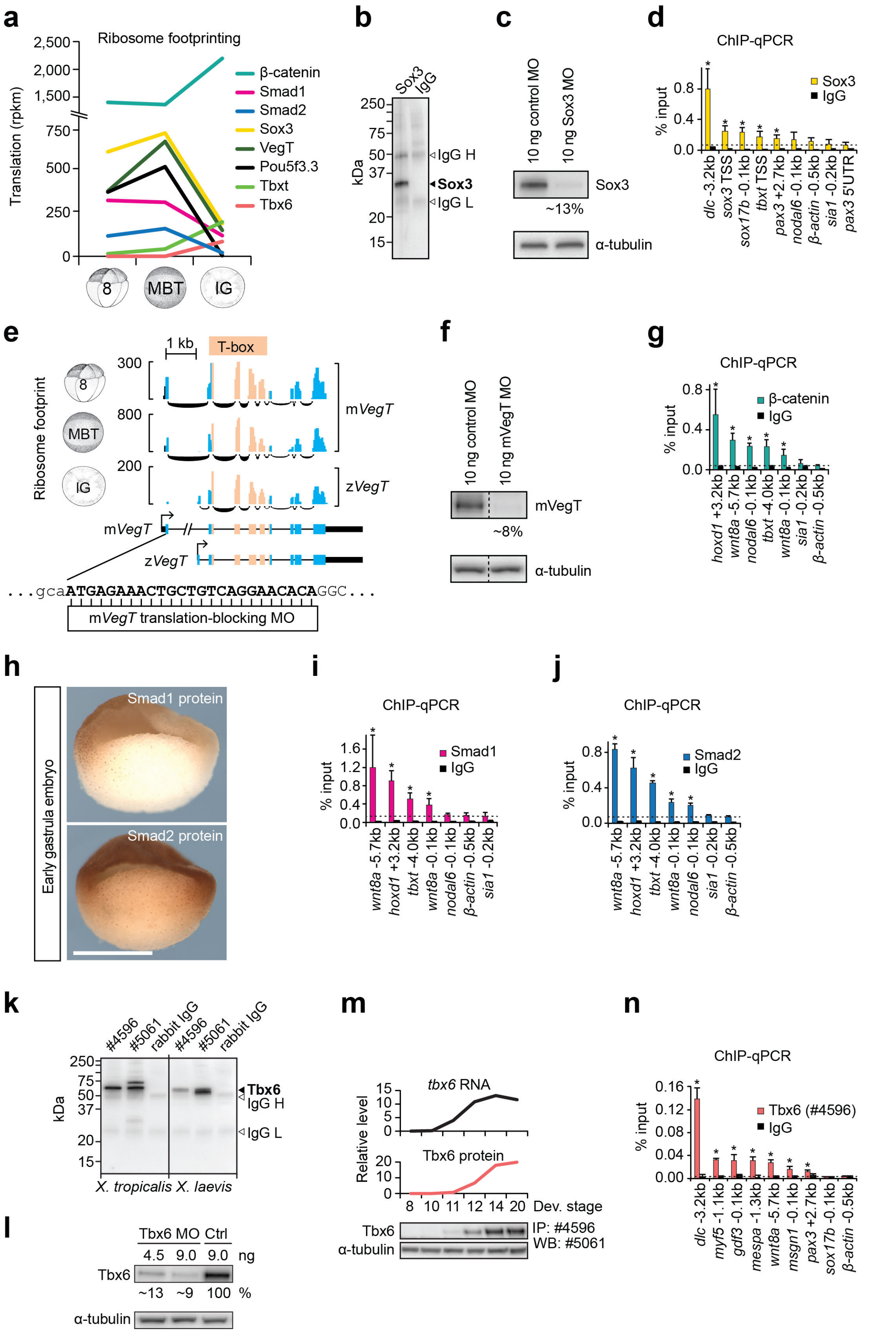

Supplementary Fig. 2 Verification of antibodies against frequently translated TFs and signal mediators. (**a**) Line chart shows the level of ribosome footprints[2] of selected TFs and signal mediators across MBT in rpkm (reads per kilobase of transcript

per million mapped reads). Abbreviations used for the developmental timeline: 8, 8-cell stage; MBT, mid-blastula transition; lG, late gastrula stage. (**b**) Western blot shows the immunoprecipitation (IP) of Sox3 protein extracted from early gastrula embryos. IgG H and L, detected IgG heavy and light chains of the IP antibody. (**c**) Western blot shows the level of Sox3 protein immunoprecipitated from control (control MO) and Sox3-depleted (Sox3 MO) blastula embryos (Sox3 LOF). α-tubulin, IP input control. (**d**,**g**,**i**,**j**,**n**) Bar graphs show the ChIP-qPCR results as a percentage of ChIP input (mean + s.d.; n=2 biologically independent samples) for Sox3, β-catenin, Smad1 and Smad2 at the early gastrula stage and for Tbx6 at the early neurula stage. One-tailed Student's t-test (comparing to IgG control): *, $p \leq 0.1$ and ≥2-fold enrichment relative to the lowest DNA recovery with the ChIP antibody (dotted line). (**e**) Ribosome footprinting[2] tracks show the post-MBT switch of translation from the maternal (m) to the zygotic (z) *VegT* transcript. The VegT translation-blocking MO was designed to block translation of the maternal transcript only. (**f**) Western blot shows the level of mVegT protein immunoprecipitated from control (control MO) and mVegT-depleted (mVegT MO) blastula embryos (mVegT LOF). Dotted line indicates the elimination of irrelevant lanes from the western blot. α-tubulin, IP input control. (**h**) WMIHC shows the spatial distribution of nuclear Smad1 and Smad2 protein on bisected early gastrula embryos. Scale bar, 0.5 mm. (**k**) Western blot shows the level of Tbx6 immunoprecipitated from *X. tropicalis* and *X. laevis* late gastrula embryos. Antibody #4596 was chosen for subsequent IP and ChIP experiments, and #5061 for Western blotting. (**l**) Western blot (WB) shows the level of Tbx6 protein immunoprecipitated from standard control and Tbx6 morphants at the early tailbud stage. α-tubulin, IP input control. (**m**) Line charts show the relative level of *tbx6* transcripts (RT-qPCR) and Tbx6 protein (IP/WB) from the early blastula to the early tailbud stage. α-tubulin, IP input control. Uncropped Western blots are shown in **Supplementary Fig. 18**.

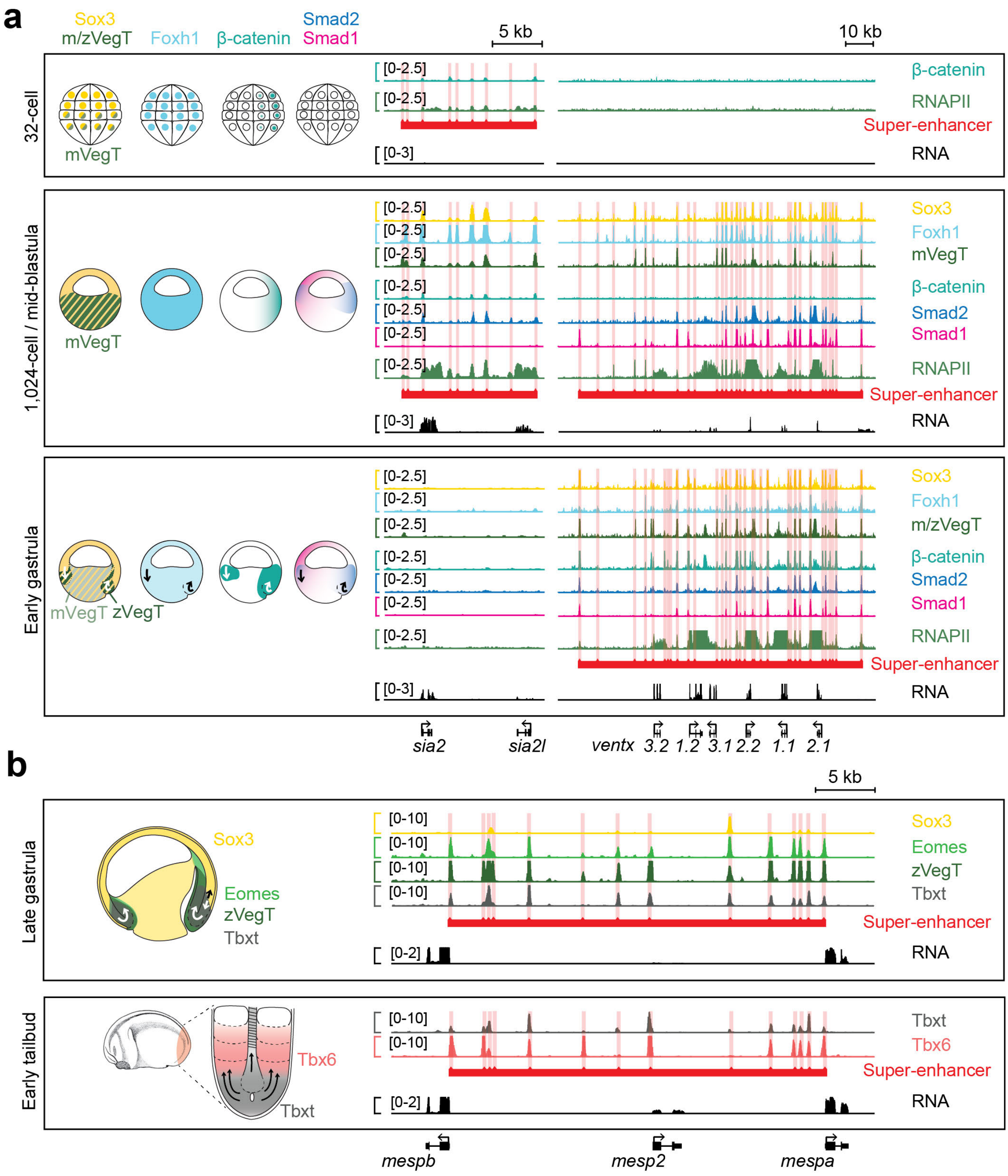

Supplementary Fig. 3 Snapshots of TF and signal mediator binding to super-enhancers during early embryogenesis. (**a**) Dynamic chromatin engagement of endogenous Sox3, Foxh1[3,4], VegT, β-catenin, Smad2, Smad1 and RNAPII to putative super-enhancers of the *siamois* and *ventx* gene cluster from the 32-cell to the early gastrula stage. Illustrated sagittal sections (dorsal side is right) show the nuclear localisation of the selected TFs with arrows pointing to the tissue movements of gastrulation. (**b**) Snapshot of endogenous Sox3, Eomes, zVegT, Tbxt and Tbx6 binding to the putative super-enhancer of the *mesp* gene cluster at the late gastrula and/or early tailbud stage. Illustrated sagittal sections (dorsal side is right) show the nuclear localisation of selected TFs in the late gastrula embryo and at the caudal end of the early tailbud embryo with arrows pointing to the tissue movements during axial elongation[5]. All super-enhancers[6] were formed by stitching together engaged pCRMs that are ≤25 kb apart.

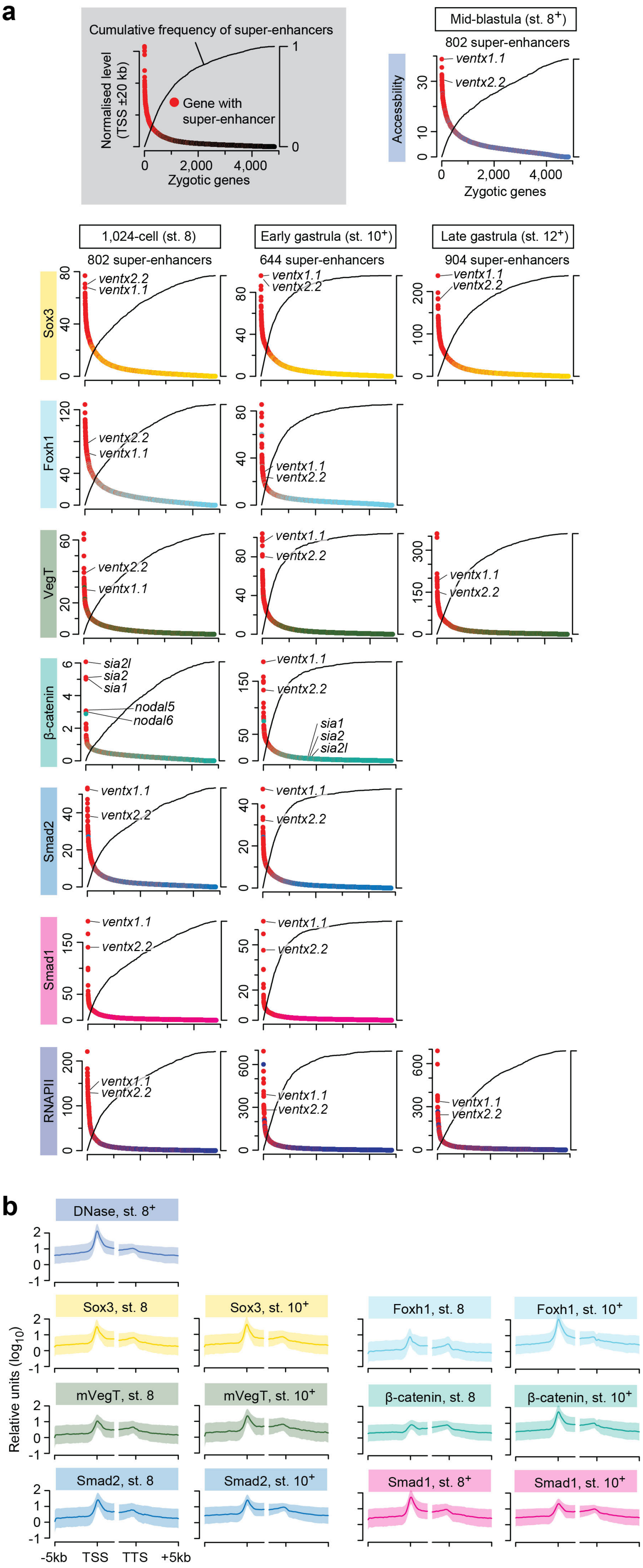

Supplementary Fig. 4 Characterisation of genome-wide chromatin engagement and chromatin accessibility in early *Xenopus* embryos. (**a**) The grey box explains the composition of the following dot plots: the x-axis displays zygotic genes (detected by RNAPII profiling[7] from the 32-cell to the late gastrula stage) ranked by the total level of pCRM accessibilities or pCRM occupancies (normalised to 1 million mapped reads; primary y-axis) that are nearest (≤20 kb) to corresponding transcription start sites (TSSs). The secondary y-axis shows the cumulative frequency of gene-associated super-enhancers (≤5 kb from TSSs). Genes with super-enhancers are highlighted in red. (**b**) Meta-summary (mean ± s.d.; $\log_{10}$ scale) of chromatin accessibility or DNA occupancy levels across zygotic TSSs and transcription termination sites (TTSs). For each developmental stage, super-enhancers were formed by stitching together pCRMs that are ≤25 kb apart and engaged by RNAPII or at least one TF or signal mediator.

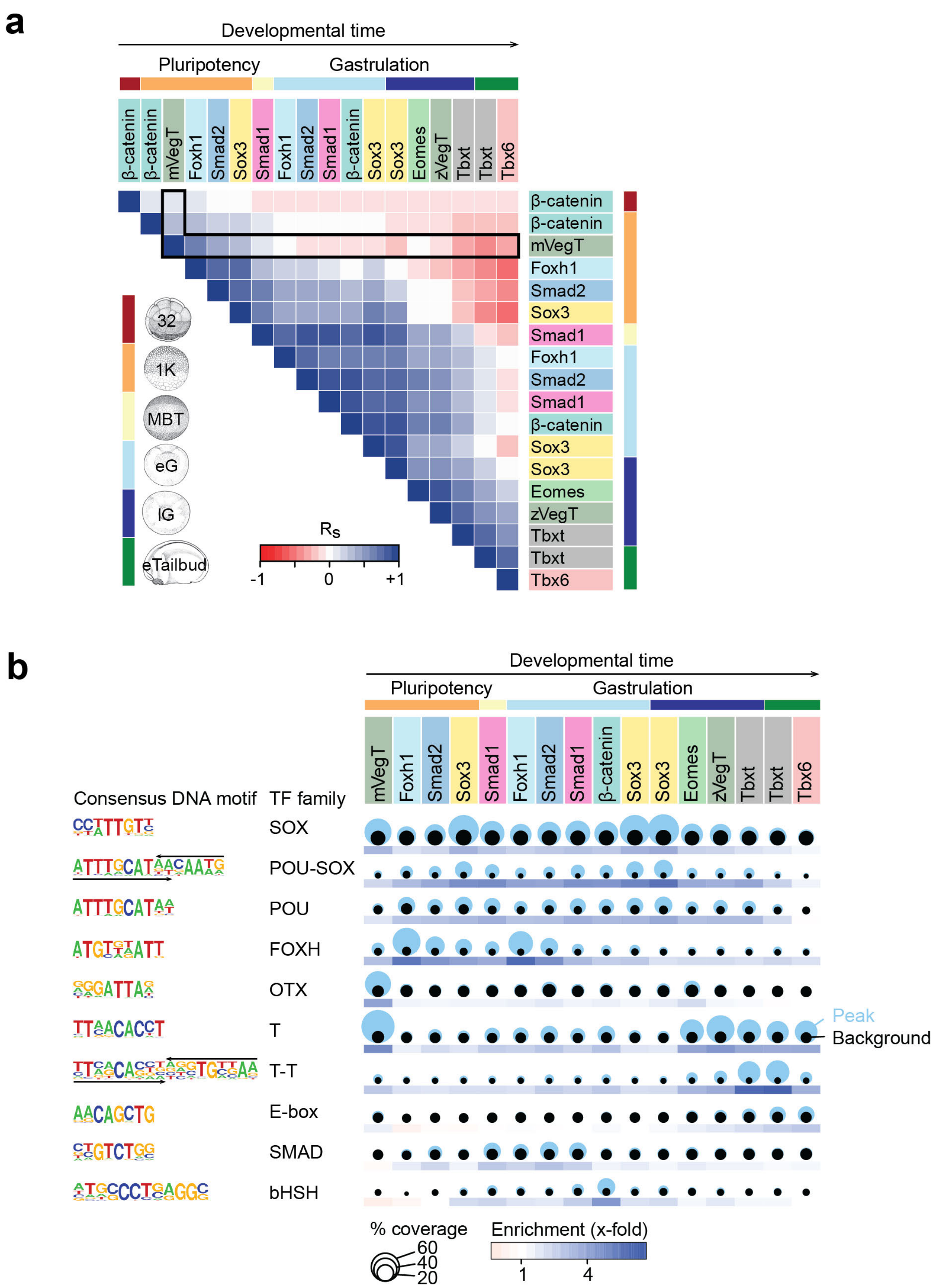

Supplementary Fig. 5 The relationship of chromatin recruitment among TFs and signal mediators and its underlying preferences for specific DNA recognition motifs in pCRMs of pluripotent and neuro-mesodermal embryonic cells. (**a**) Heat map shows pairwise Spearman correlations (R_s) of DNA occupancy levels at ~12,500 pCRMs among TFs (Sox3, mVegT, Foxh1[3,4], Eomes[8], zVegT[8], Tbxt[8] and Tbx6) and signal mediators (β-catenin, Smad1, Smad2) at indicated developmental stages. The pCRM coordinates were generated by collating the strongest 2,000 peaks of each binding profile. Abbreviations used for the developmental timeline: 32 and 1K, 32- and 1,024-cell stage; MBT, mid-blastula transition; eG and lG, early and late gastrula stage; eTailbud, early tailbud stage. (**b**) Bubble plots and heat maps show the coverage and enrichment of DNA recognition motifs, respectively, among the top 2,000 pCRMs in each chromatin profile.

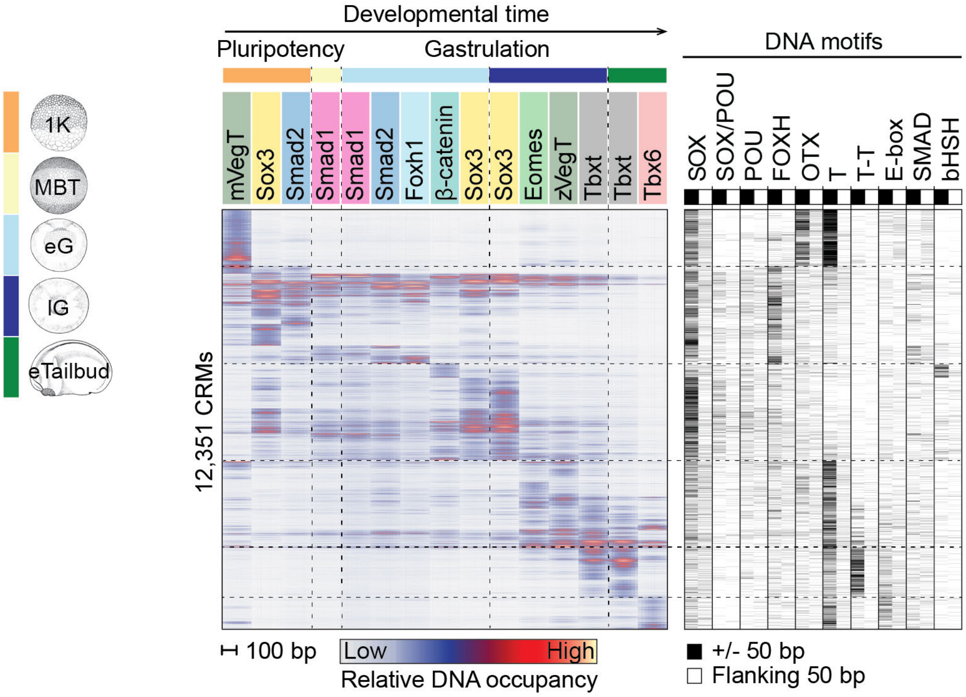

Supplementary Fig. 6 pCRM binding patterns correlate with the occurrence of specific DNA motifs. Heat map to the left shows the relative DNA occupancy levels across ~12,500 pCRMs for a selection of TFs (mVegT, Sox3, Foxh1[3,4], Eomes[8], zVegT[8], Tbxt[8] and Tbx6) and signal mediators (β-catenin, Smad1, Smad2) from the 1,024-cell to the early tailbud stage. pCRM were hierarchically clustered according to the binding levels of all TFs and signal mediators. The pCRM coordinates were generated by collating the strongest 2,000 peaks of each binding profile. Heat map to the right shows the occurrence of specific DNA motifs ±50 bp from the pCRM centre as well as the flanking 50 bp. The consensus sequences of these DNA motifs are shown in **Supplementary Fig. 5b**. Abbreviations used for the developmental timeline: 1K, 1,024-cell stage; MBT, mid-blastula transition; eG and lG, early and late gastrula stage; eTailbud, early tailbud stage.

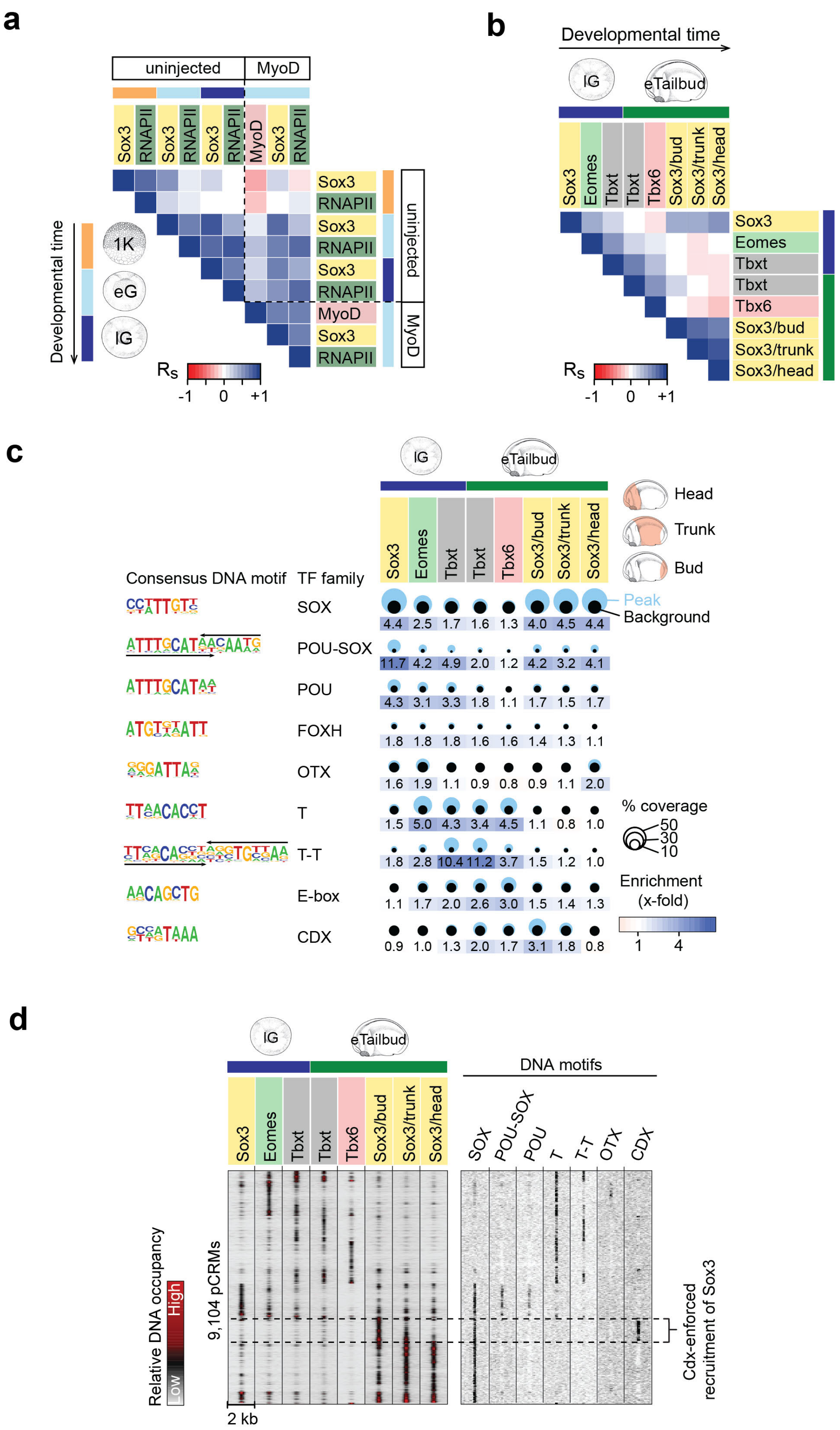

Supplementary Fig. 7 TF co-expression influences chromatin engagement. (**a**,**b**) Heat maps show Spearman correlations (R_s) of DNA occupancy levels of indicated factors in different developmental contexts. (**c**) Bubble plot and heat map show the

respective coverage and enrichment of DNA motifs in pCRMs bound by Sox3, Eomes[8], Tbxt[8] and Tbx6 in late gastrula and early tailbud embryos. For Sox3 profiling, the embryo was dissected into three parts: head, trunk and tailbud. (**d**) Heat map shows the hierarchical clustering of 9,104 pCRMs according to relative DNA occupancy levels. The pCRM coordinates were generated by collating the strongest 2,000 peaks of each binding profile. Abbreviations of the developmental timeline: 1K, 1,024-cell (early blastula); eG, early gastrula; lG, late gastrula; and eTailbud, early tailbud.

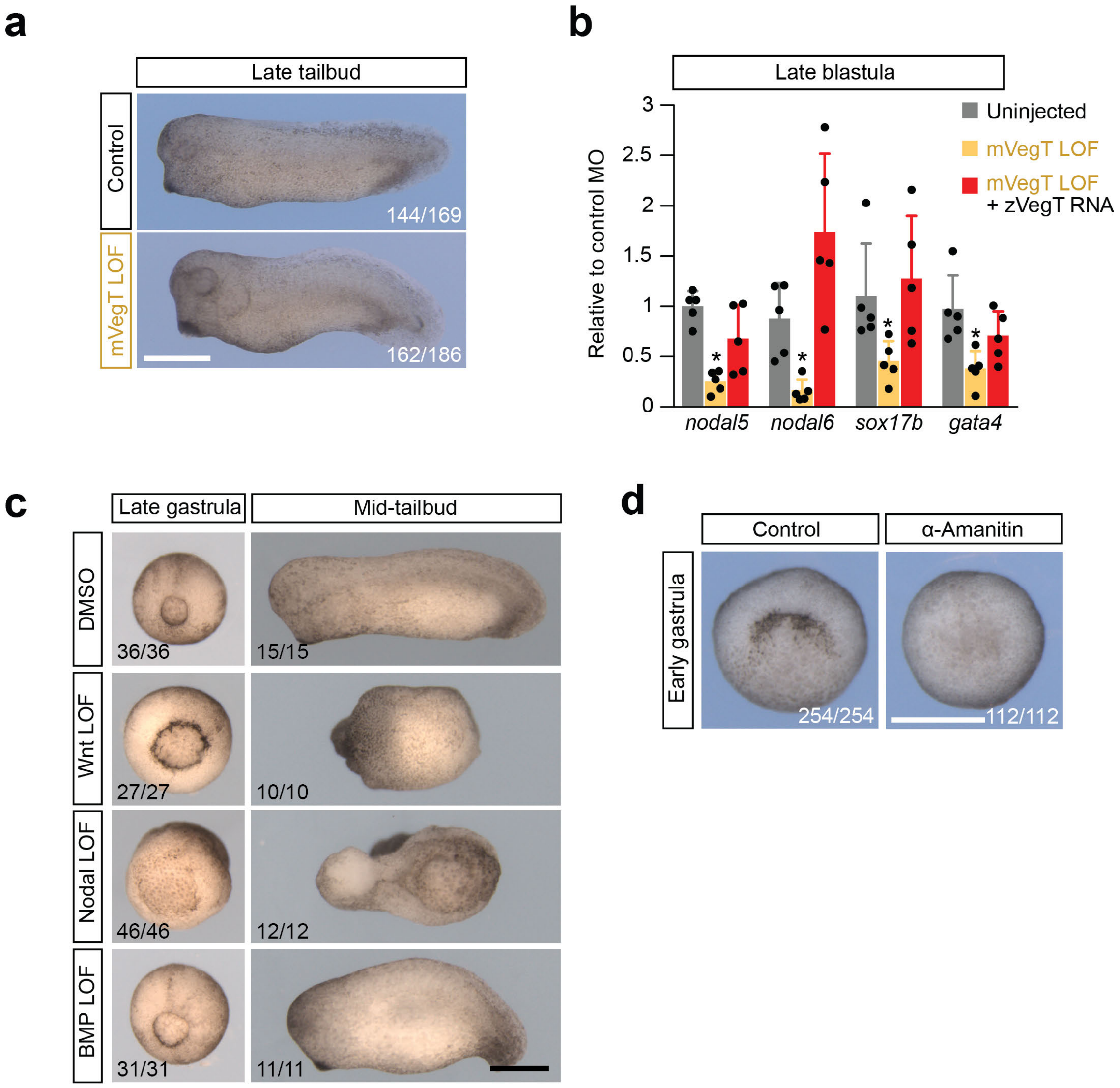

Supplementary Fig. 8 Morphological defects caused by the functional loss of maternal TFs and signal mediators (**a**) Morphological phenotype caused by the LOF of mVegT at the late tailbud stage. (**b**) Bar graph shows the RT-qPCR results of rescuing reduced *nodal5*, *nodal6*, *sox17b* and *gata4* transcript levels in mVegT LOF embryos by the injection of *X. tropicalis* z*VegT* mRNA. Error bars, mean + s.d. (n=5 biologically independent samples). Two-tailed Student's t-test: *, p <0.02. (**c**) Morphological phenotypes caused by the single LOF of canonical Wnt, Nodal or BMP signalling when control embryos reached the late gastrula and mid-tailbud stage. (**d**) Morphological phenotype caused by the injection of α-amanitin when control embryos reached the early gastrula stage. Numbers in the right or left bottom corner of each image refer to the count of embryos detected with the displayed morphological phenotype. Scale bars, 0.5 mm.

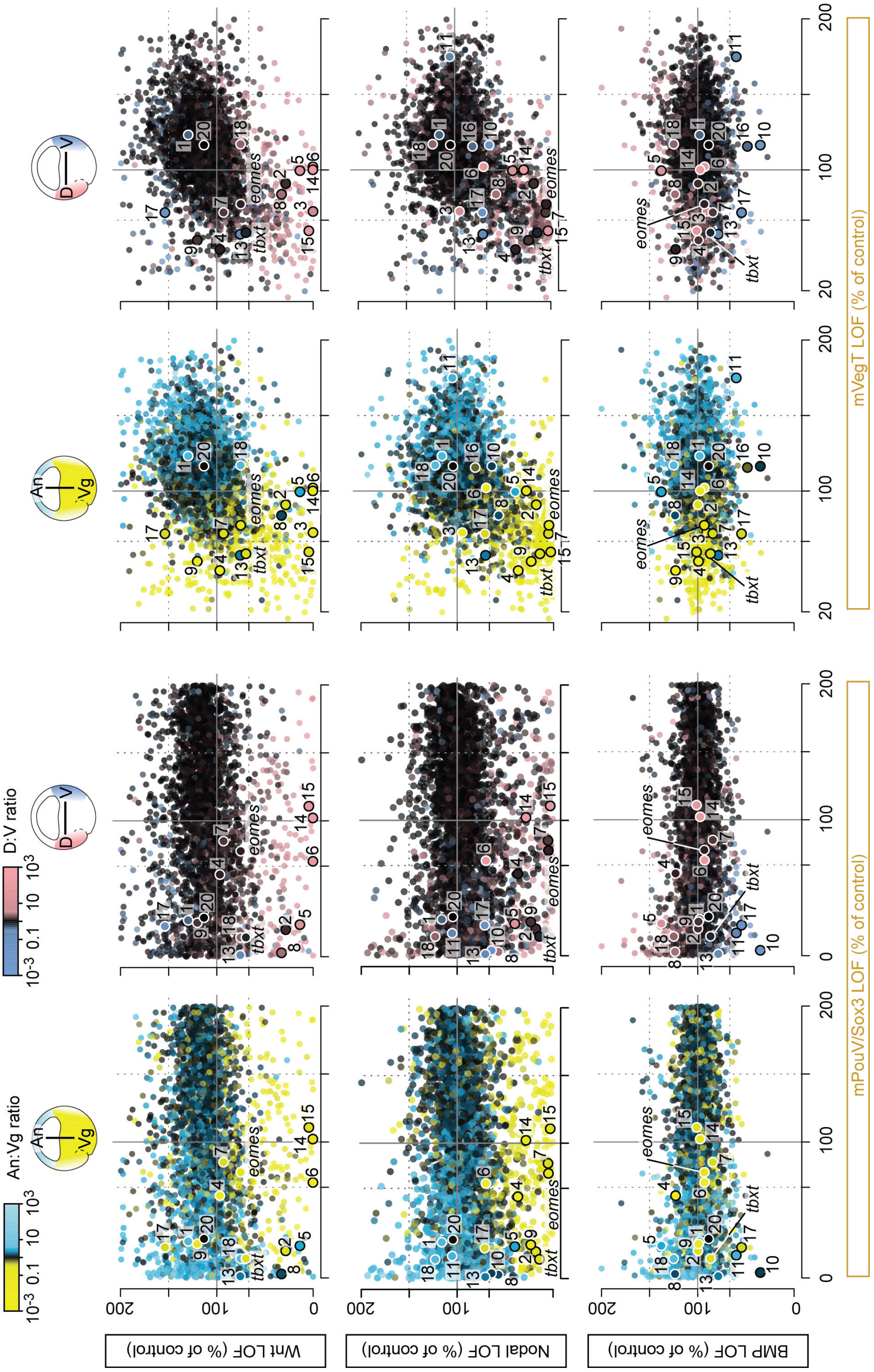

Supplementary Fig. 9 Transcriptional comparison of zygotic genes between indicated LOFs of maternal TFs or signals. Dots are colored according to the normal ratio of transcript levels (regional expression[9]) across the animal-vegetal (An:Vg) or dorso-ventral (D:V) axis. Numbered dots refer to genes listed in **Fig. 6e**.

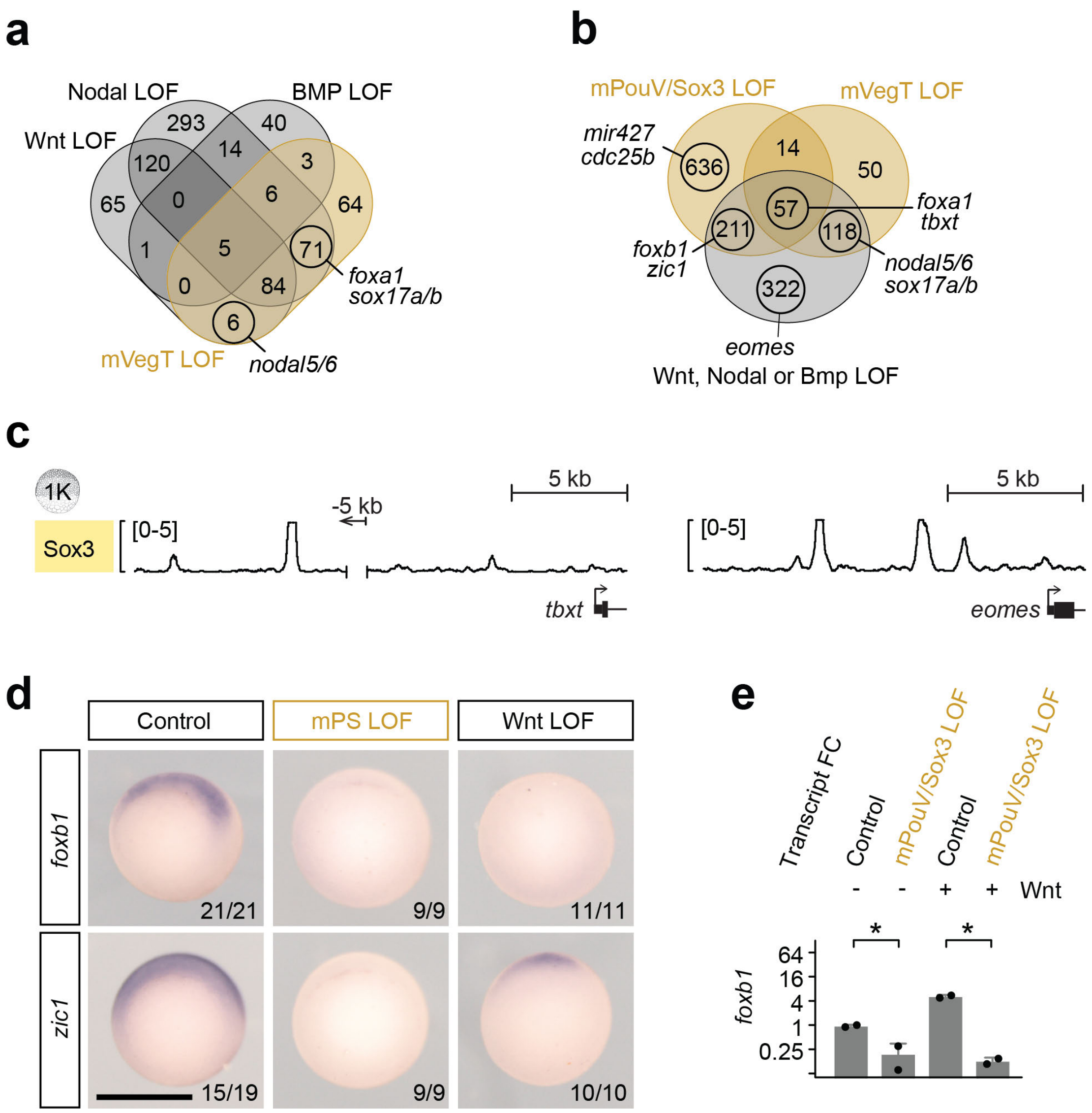

Supplementary Fig. 10 Signal-induced regionalisation of ZGA depends on maternal TFs. (**a**,**b**) Venn diagrams show the number of genes downregulated by indicated LOFs. (**c**) ChIP-Seq track of Sox3 binding to the genomic loci of *tbxt* and *eomes* at the 1,024-cell stage (1K). (**d**) Early gastrula-staged WMISHs show that Wnt-induced transcription of *foxb1* and *zic1* on the dorsal side of the embryo depends on ubiquitously expressed maternal pluripotency factors mPouV and Sox3 (mPS). Embryos are imaged from the vegetal side and orientated so that dorsal side faces top. Numbers in the right bottom corner of each image refer to the count of embryos detected with the displayed WMISH staining among all embryos analysed per condition and *in situ* probe. Scale bar, 0.5 mm. (**e**) Bar graph shows the relative quantification of *foxb1* transcript levels (RT-qPCR) in control and mPouV/Sox3 LOF animal caps with or without canonical Wnt signalling. Error bars, mean + s.d. (n=2 biologically independent samples). Two-tailed Student's t-test: *, p=0.05.

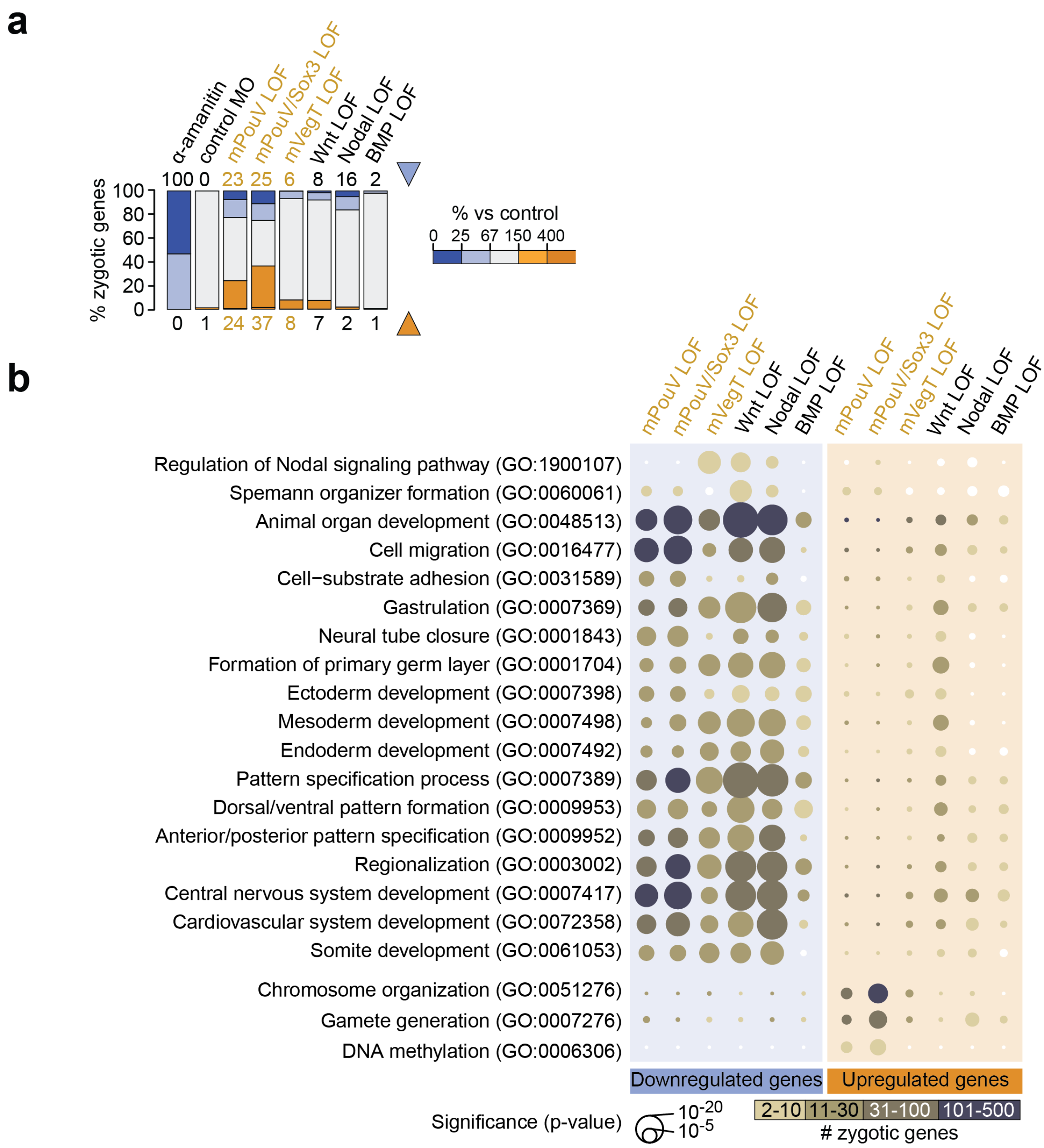

Supplementary Fig. 11 Selected maternal TFs and inductive signals regulate the regionalisation of ZGA and the formation of primary body axes and germ layers. (**a**) Stacked bar graph shows the percentage of zygotic genes misregulated by indicated LOFs. Numbers above and below the bars indicate the percentage of downregulated and upregulated genes, respectively. (**b**) Bubble plot shows the enrichment of key GO term-related biological processes among the zygotic genes misregulated by indicated LOFs. Size of bubble reflects the statistical significance (hypergeometric p-value) of enrichment while the color indicates the number of affected genes.

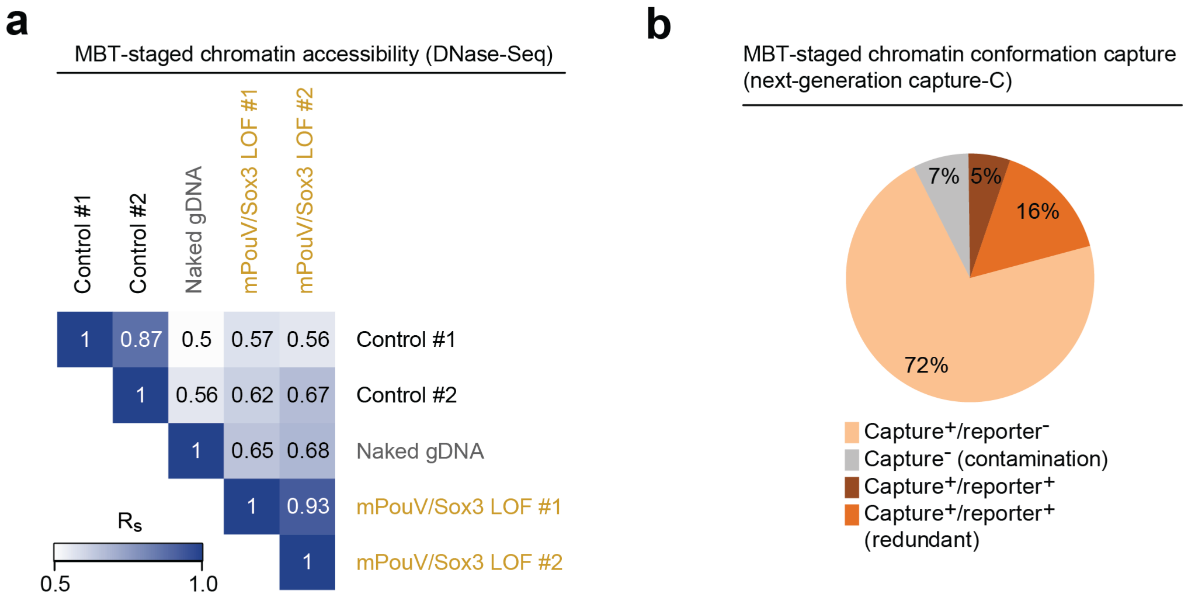

Supplementary Fig. 12 Performance of DNase-Seq and next-generation capture-C. (**a**) Heat map shows pairwise Spearman correlations (R_s) among indicated biological replicates of MBT-staged DNase-Seq on extracted chromatin and naked genomic DNA (gDNA). (**b**) Pie chart summarises the sequence composition (capture/reporter) of FLASH[10] reads. Promoter contacts (capture) with distal genomic elements (reporter) were enriched by two rounds of hybridisation with promoter-specific probes (see **Fig. 9b** and **Supplementary Data 10**).

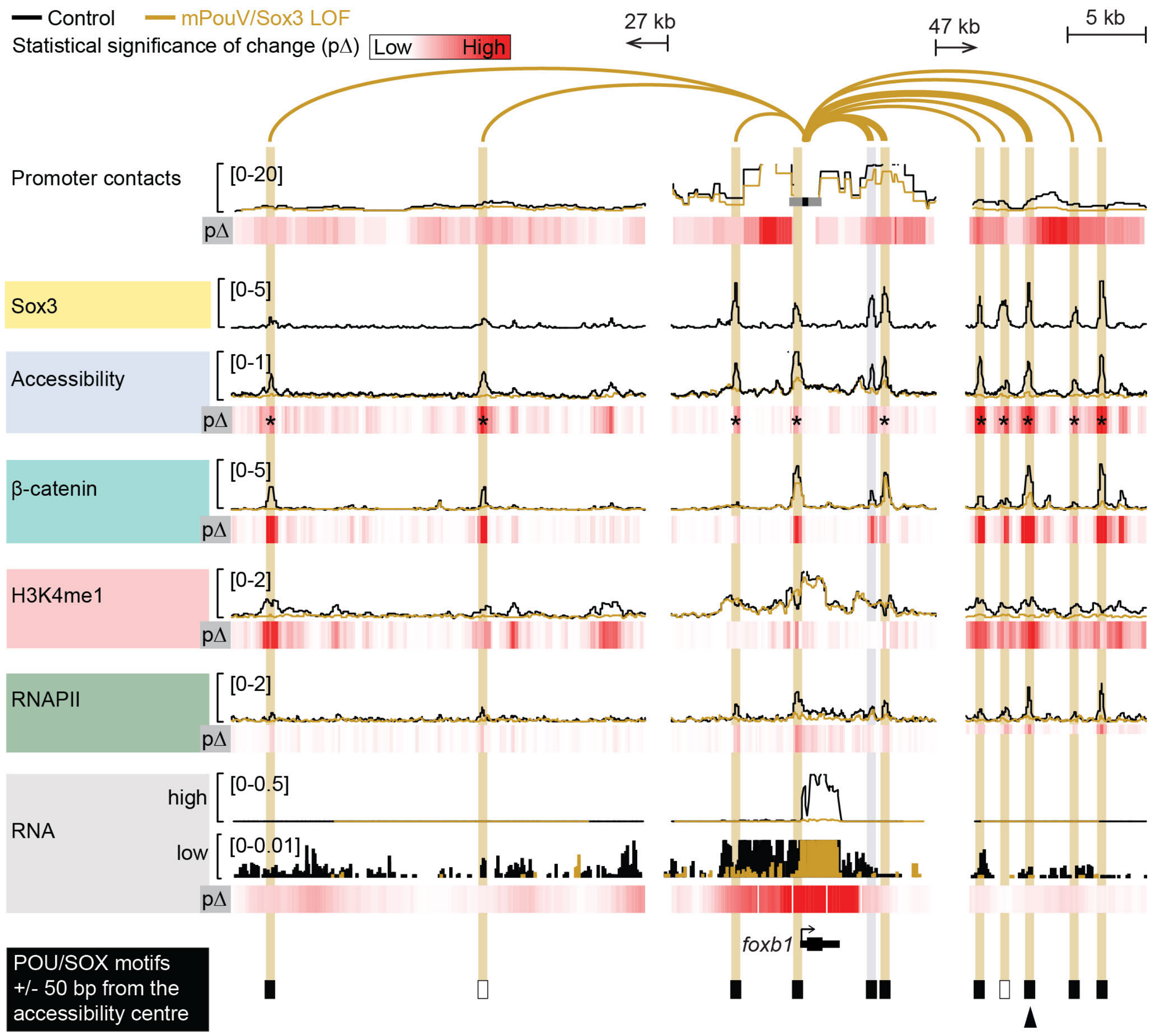

Supplementary Fig. 13 Pioneering activity of maternal PouV/Sox3 initiates extensive chromatin remodelling. Superimposed line tracks show promoter contact frequencies, chromatin accessibilities and DNA occupancies of various chromatin components (β-catenin, H3K4me1 and RNAPII) at the *foxb1* gene locus between control (uninjected) and mPouV/Sox3 LOF embryos. The RNA track is split into a high (0-0.5) and low (0-0.01) expression window. Note that the low-expression window shows that both transcription of local non-coding super-enhancer RNA and the gene *foxb1* depend on mPouV/Sox3. Heat maps (pΔ) below each superimposed line plot show the statistical significance (Wald test) of changes caused by mPouV/Sox3 LOF. The footer highlights the occurrences of canonical POU/SOX motifs (black filled rectangles) at accessible pCRMs (±50 bp from the accessibility centre) and one strongly affected pCRM with an arrowhead. Asterisks on the pΔ heat map mark significant (FDR ≤10%) reductions to pCRM accessibility. pCRMs are boxed in and their frequency of contacts with the *foxb1* promoter are illustrated with an arc of varying strength. Boxes of affected pCRM and arcs of promoter contacts are colored orange.

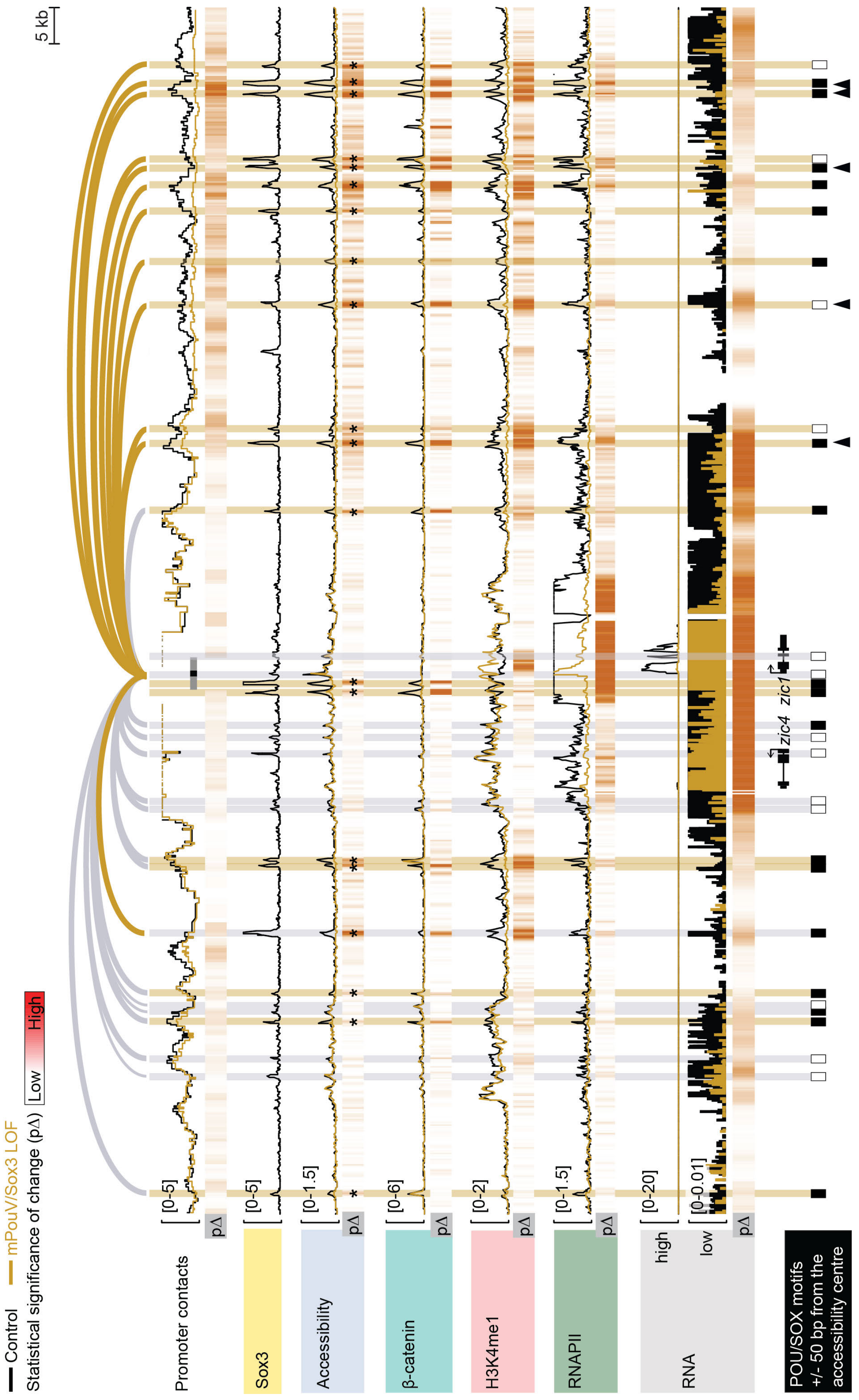

Supplementary Fig. 14 Pioneering activity of maternal PouV/Sox3 initiates extensive chromatin remodelling. Superimposed line tracks show promoter contact frequencies, chromatin accessibilities and DNA occupancies of various chromatin

components (β-catenin, H3K4me1 and RNAPII) at the *zic1* gene locus between control (uninjected) and mPouV/Sox3 LOF embryos. The RNA track is split into a high (0-20) and low (0-0.01) expression window. Note that the low-expression window shows that both transcription of local non-coding super-enhancer RNA and the gene *zic1* depend on mPouV/Sox3. Heat maps (pΔ) below each superimposed line plot show the statistical significance (Wald test) of changes caused by mPouV/Sox3 LOF. The footer highlights the occurrences of canonical POU/SOX motifs (black filled rectangles) at accessible pCRMs (±50 bp from the accessibility centre) and strongly affected pCRMs with arrowheads. Asterisks on the pΔ heat map mark significant (FDR ≤10%) reductions to pCRM accessibility. pCRMs are boxed in and their frequency of contacts with the *zic1* promoter are illustrated with an arc of varying strength. Boxes of affected pCRMs and arcs of promoter contacts are colored orange.

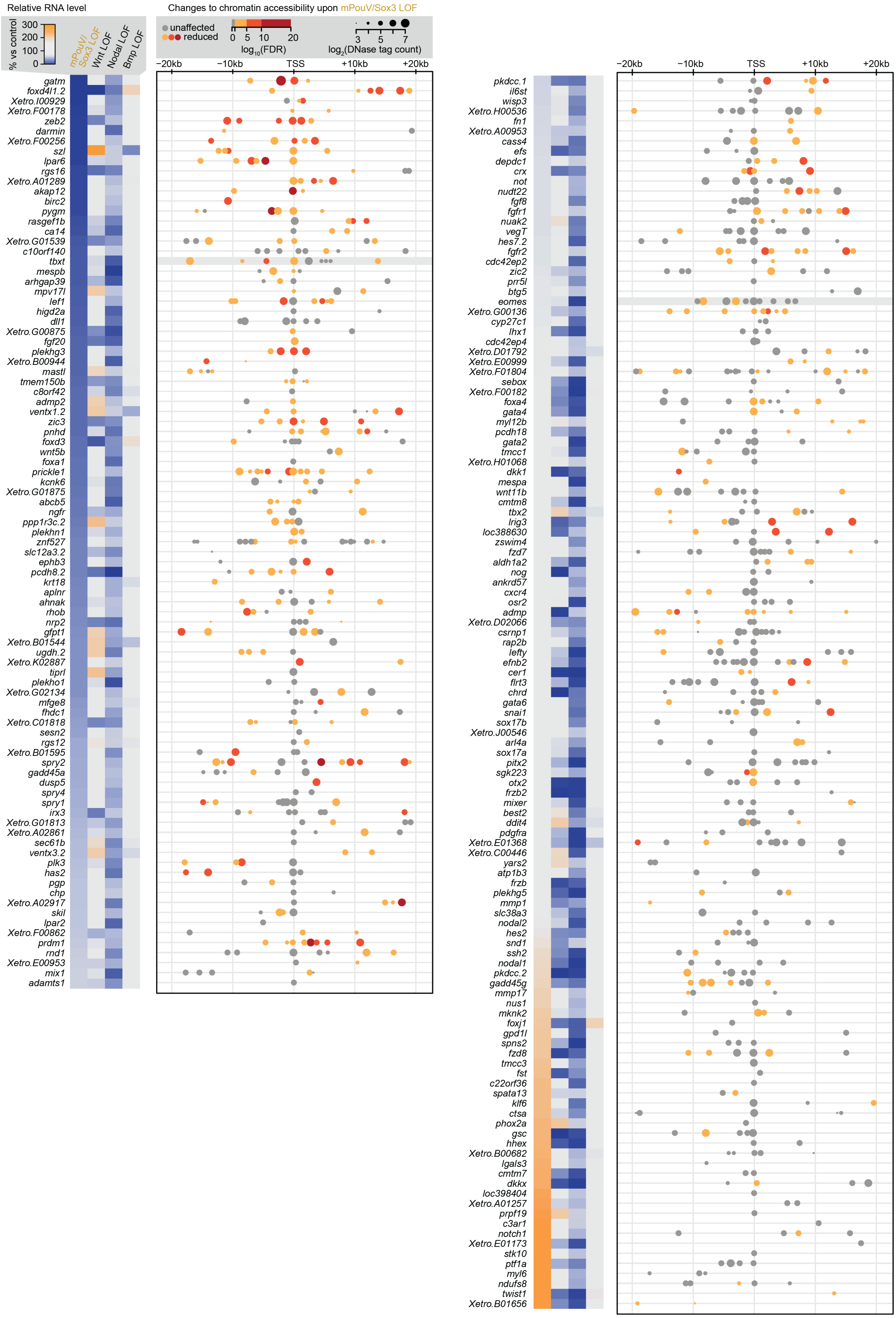

Supplementary Fig. 15 mPouV/Sox3-induced chromatin accessibility is required for the expression of Nodal-responsive genes. The heat map shows the transcript levels of Nodal-responsive genes under indicated LOFs. All genes listed here are

active by the MBT[7] and their transcript levels are significantly reduced (≥two-fold; FDR ≤10%) upon α-amanitin injection (see **Fig. 6e**). The plot aligned to the heat map shows the localisation and DNase sensitivity (bubble size) of accessible pCRMs (affected, dot colored in orange to red with FDR decreasing from 10%; and unaffected, grey dot) relative to zygotic TSSs. Gene loci are sorted by mPouV/Sox3 LOF-induced transcript fold changes as shown in the heat map.

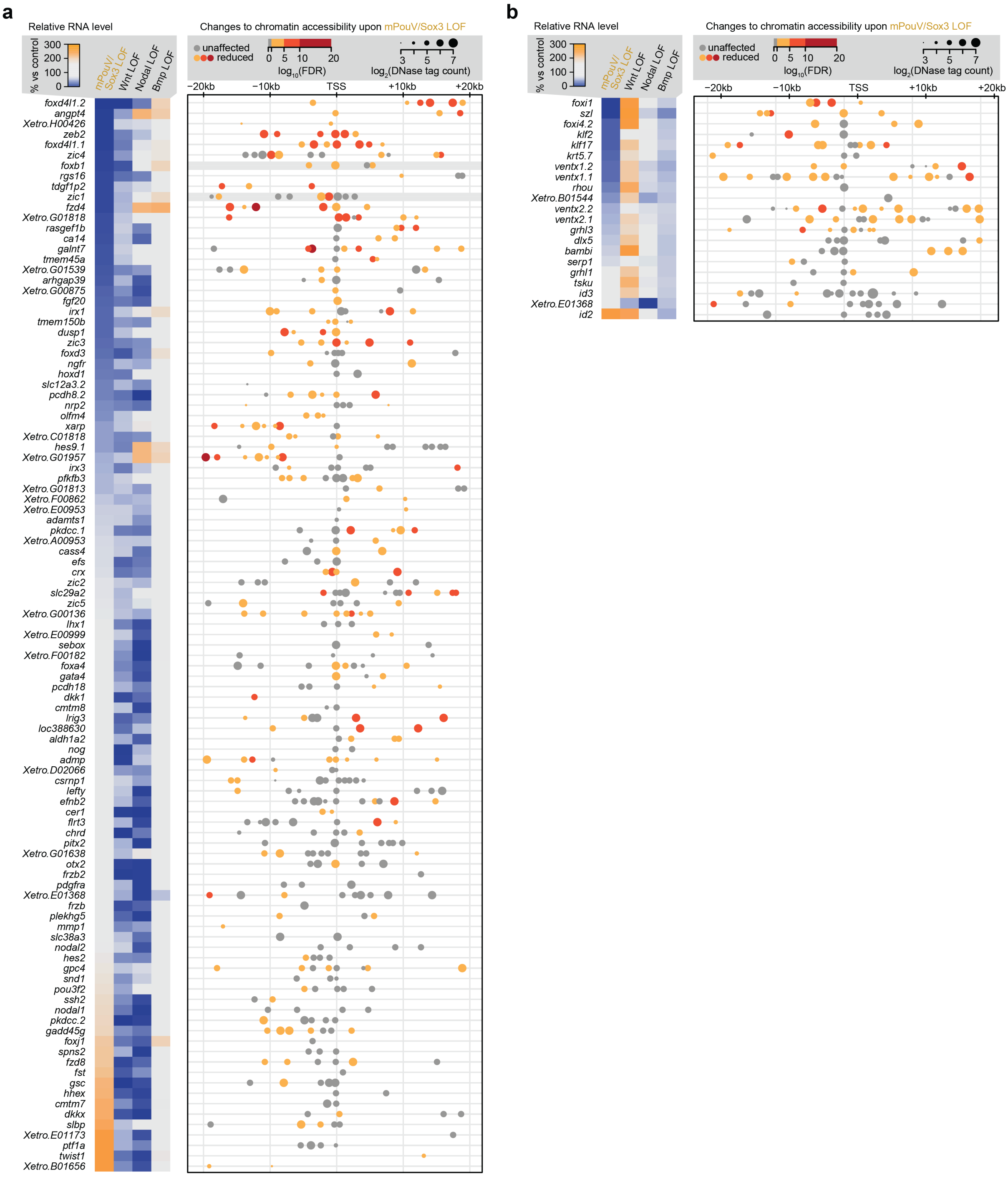

Supplementary Fig. 16 mPouV/Sox3-induced chromatin accessibility is required for the expression of Wnt- and BMP responsive genes. (**a**,**b**) Heat maps show the transcript levels of Wnt- or Bmp-responsive genes under indicated LOFs. All genes listed here are active by the MBT[7] and their transcript levels are significantly reduced (≥two-fold; FDR ≤10%) upon α-amanitin injection (see **Fig. 6e**). The plot aligned to the heat map shows the localisation and DNase sensitivity (bubble size) of accessible pCRMs (affected, dot colored in orange to red with FDR decreasing from 10%; and unaffected, grey dot) relative to zygotic TSSs. Gene loci are sorted by mPouV/Sox3 LOF-induced transcript fold changes as shown in the heat map.

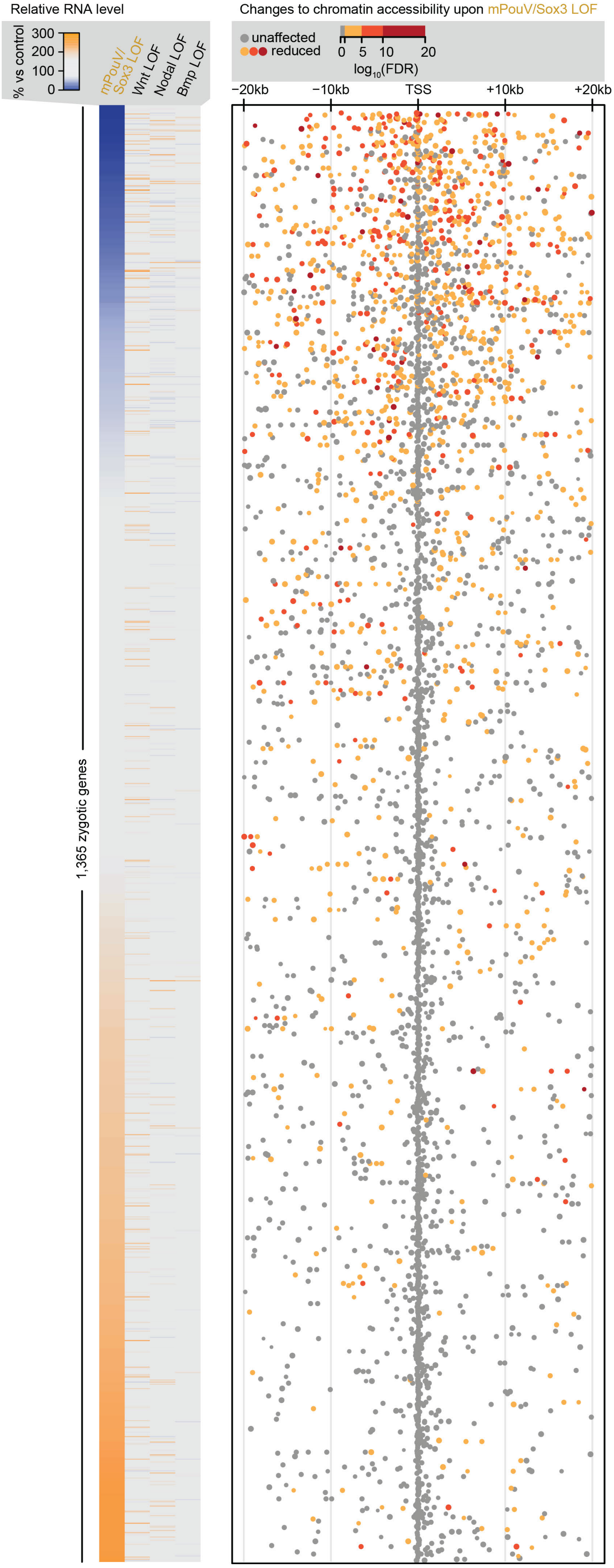

Supplementary Fig. 17 mPouV/Sox3-induced chromatin accessibility is required for the expression of signal non-responsive genes. The heat map shows the transcript levels of 1,365 signal non-responsive genes under indicated LOFs. The plot aligned to the heat map shows the localisation of accessible pCRMs (affected, dot colored in orange to red with FDR decreasing from 10%; and unaffected, grey dot) relative to the zygotic TSSs. All genes listed here are active by the MBT[7] and their transcript levels are significantly reduced (≥two-fold; FDR ≤10%) upon α-amanitin injection (see **Fig. 6e**). Gene loci are sorted by mPouV/Sox3 LOF-induced transcript fold changes as shown in the heat map.

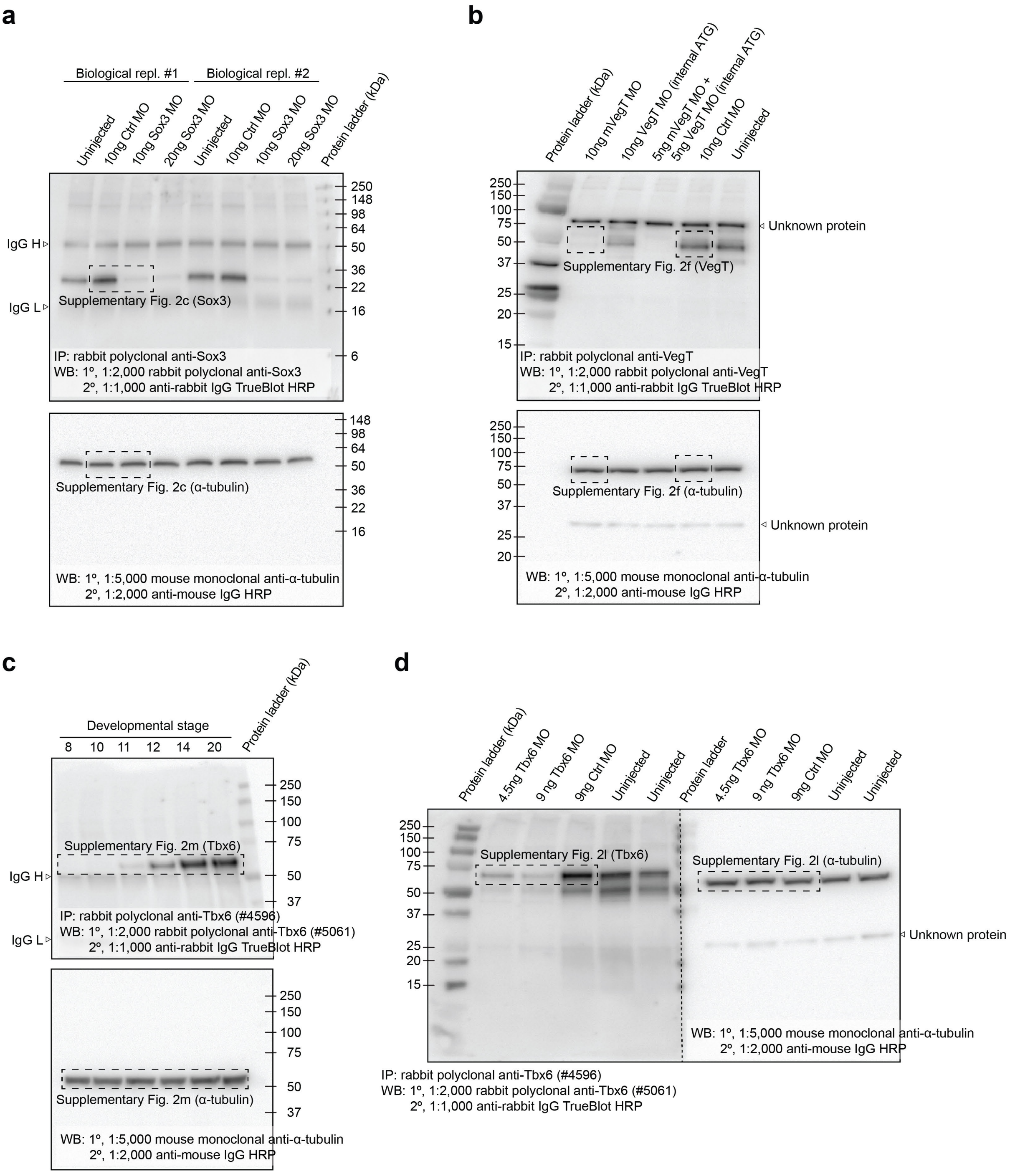

Supplementary Fig. 18 Uncropped Western blots. Dotted rectangles outline the sections shown in the Supplementary Fig. 2. (**a**) Western blot shows the level of Sox3 protein immunoprecipitated from control (uninjected, control MO) and Sox3-depleted (10ng or 20ng Sox3 MO) blastula embryos (Sox3 LOF). IgG H and L, detected IgG heavy and light chains of the immunoprecipitation (IP) antibody. α-tubulin, IP input control. (**b**) Western blot shows the level of mVegT protein immunoprecipitated from control (uninjected, control MO, inefficient VegT MO targeting an internal ATG, 5'-CTTGGCTGGAGACGCTGTCCATATC-3') and mVegT-depleted (mVegT MO) blastula embryos (mVegT LOF). α-tubulin, IP input control. (**c**) Western blot shows the level of Tbx6 immunoprecipitated from *X. tropicalis* embryos over several developmental stages from the early blastula to the early tailbud stage. α-tubulin, IP input control. (**d**) Western blot shows the level of Tbx6 protein immunoprecipitated from uninjected embryos and standard control and Tbx6 morphants at the early tailbud stage. α-tubulin, IP input control.

Supplementary Notes

Supplementary Note 1 Percentage of TF^+ or signal $mediator^+$ pCRMs bound (ChIP ≥2x input tag density) by RNAPII (**Fig. 3c**): 98% (1,024-cell stage) and 99% (early gastrula stage) of $Sox3^+$ pCRMs, 100% and 90% of $Foxh1^+$ pCRMs, 93% and 97% of $VegT^+$ pCRMs, 65% and 98% of β-$catenin^+$ pCRMs, 96% and 98% of $Smad2^+$ pCRMs, and 78% and 99% of $Smad1^+$ pCRMs. Percentage of TF^+ pCRMs bound (ChIP ≥2x input tag density) by signal mediators (**Fig. 3f**): β-catenin at $Sox3^+$ pCRMs (10% at the 1,024-cell stage and 100% at the early gastrula stage), Smad2 at $Sox3^+$ pCRMs (96% and 91%), Smad1 at $Sox3^+$ pCRMs (96% and 85%), β-catenin at $Foxh1^+$ pCRMs (12% and 87%), Smad2 at $Foxh1^+$ pCRMs (94% and 82%), Smad1 at $Foxh1^+$ pCRMs (63% and 59%), β-catenin at $VegT^+$ pCRMs (11% and 100%), Smad2 at $VegT^+$ pCRMs (64% and 92%) and Smad1 at $VegT^+$ pCRMs (55% and 68%).

Supplementary References

1. Hontelez, S. *et al.* Embryonic transcription is controlled by maternally defined chromatin state. *Nat. Commun.* **6,** 10148 (2015).
2. Subtelny, A. O., Eichhorn, S. W., Chen, G. R., Sive, H. & Bartel, D. P. Poly(A)-tail profiling reveals an embryonic switch in translational control. *Nature* **508,** 66–71 (2014).
3. Charney, R. M. *et al.* Foxh1 Occupies cis-Regulatory Modules Prior to Dynamic Transcription Factor Interactions Controlling the Mesendoderm Gene Program. *Dev. Cell* **40,** 595–607.e4 (2017).
4. Chiu, W. T. *et al.* Genome-wide view of TGF /Foxh1 regulation of the early mesendoderm program. *Development* **141,** 1–114 (2014).
5. Gentsch, G. E., Monteiro, R. S. & Smith, J. C. Cooperation Between T-Box Factors Regulates the Continuous Segregation of Germ Layers During Vertebrate Embryogenesis. *Curr. Top. Dev. Biol.* **122,** 117–159 (2017).
6. Whyte, W. A. *et al.* Master transcription factors and mediator establish super-enhancers at key cell identity genes. *Cell* **153,** 307–319 (2013).
7. Gentsch, G. E., Owens, N. D. L. & Smith, J. C. The Spatiotemporal Control of Zygotic Genome Activation. *iScience* **16,** 485–498 (2019).
8. Gentsch, G. E. *et al.* In vivo T-box transcription factor profiling reveals joint regulation of embryonic neuromesodermal bipotency. *Cell Rep.* **4,** 1185–1196 (2013).
9. Blitz, I. L. *et al.* A catalog of Xenopus tropicalis transcription factors and their regional expression in the early gastrula stage embryo. *Dev. Biol.* **426,** 409–417 (2017).
10. Magoč, T. & Salzberg, S. L. FLASH: fast length adjustment of short reads to improve genome assemblies. *Bioinformatics* **27,** 2957–2963 (2011).
