# Supplementary material for: Maternal pluripotency factors initiate extensive chromatin remodelling to predefine first response to inductive signals
Source: Nat Commun. 2019 Sep 19;10:4269. doi: 10.1038/s41467-019-12263-w (PMC6753111; doi:10.1038/s41467-019-12263-w)
Supplement: Supplementary file 3 — Description of Additional Supplementary Files [file 41467_2019_12263_MOESM3_ESM.pdf]

Description of Additional Supplementary Files

File Name: Supplementary Data 1

Description: Summary of deep sequencing and read alignments

File Name: Supplementary Data 2

Description: Annotation of RNAPII+pCRMs (genomic coordinates, distance to nearest TSS, DNA occupancy levels from the 32-cell to the late gastrula stage, DNA motif counts)

File Name: Supplementary Data 3

Description: Annotation of accessible pCRMs (genomic coordinates, distance to nearest TSS, normalised DNase cleavage count and DNA occupancy levels of RNAPII and H3K4me1)

File Name: Supplementary Data 4

Description: Maternal protein concentrations versus ribosome densities (translation) across the mid-blastula transition

File Name: Supplementary Data 5

Description: ChIP-Seq peak calling coordinates for TFs (Eomes, Foxh1, Sox3, Tbx6, Tbxt and VegT) and signal mediators (catenin, Smad1 and Smad2) at indicated developmental stages

File Name: Supplementary Data 6

Description: Genomic coordinates of super-enhancers assembled from individual TF and signal mediator ChIP-Seq profiles separated by developmental stages (up to MBT, early gastrula stage and late gastrula stage)

File Name: Supplementary Data 7

Description: ChIP-Seq peak calling coordinates for Sox3 and MyoD-HA in early gastrula embryos with ectopic expression of MyoD-HA

File Name: Supplementary Data 8

Description: ChIP-Seq peak calling coordinates for Sox3 in dissected parts of the early tailbud embryo (head, trunk and bud)

File Name: Supplementary Data 9

Description: Transcriptional effect of maternal transcription factors and inductive signals on ZGA

File Name: Supplementary Data 10

Description: Genomic coordinates of selected capture-C viewpoints and DNA sequences used for capturing promoter contacts

File Name: Supplementary Data 11

Descriptiom: MBT-staged chromatin accessibility changes caused by mPouV/Sox3 LOF

File Name: Supplementary Data 12

Description: Results of the next-generation capture-C performed at the MBT on control and mPouV/Sox3 LOF embryos

File Name: Supplementary Movie 1

Description: Gastrulation defects caused by mPouV/Sox3 LOF
