# Supplementary material for: Maternal pluripotency factors initiate extensive chromatin remodelling to predefine first response to inductive signals
Source: Nat Commun. 2019 Sep 19;10:4269. doi: 10.1038/s41467-019-12263-w (PMC6753111; doi:10.1038/s41467-019-12263-w)
Supplement: Supplementary file 17 — Reporting Summary [file 41467_2019_12263_MOESM17_ESM.pdf]

natureresearch

Corresponding author(s): George E. Gentsch & James C. Smith

Reporting Summary

Nature Research wishes to improve the reproducibility of the work that we publish. This form provides structure for consistency and transparency in reporting. For further information on Nature Research policies, see Authors & Referees and the Editorial Policy Checklist.

Statistical parameters

When statistical analyses are reported, confirm that the following items are present in the relevant location (e.g. figure legend, table legend, main text, or Methods section).

n/a	Confirmed	
☐	☒	The exact sample size (*n*) for each experimental group/condition, given as a discrete number and unit of measurement
☐	☒	An indication of whether measurements were taken from distinct samples or whether the same sample was measured repeatedly
☐	☒	The statistical test(s) used AND whether they are one- or two-sided<br>*Only common tests should be described solely by name; describe more complex techniques in the Methods section.*
☒	☐	A description of all covariates tested
☐	☒	A description of any assumptions or corrections, such as tests of normality and adjustment for multiple comparisons
☐	☒	A full description of the statistics including central tendency (e.g. means) or other basic estimates (e.g. regression coefficient) AND variation (e.g. standard deviation) or associated estimates of uncertainty (e.g. confidence intervals)
☐	☒	For null hypothesis testing, the test statistic (e.g. *F*, *t*, *r*) with confidence intervals, effect sizes, degrees of freedom and *P* value noted<br>*Give P values as exact values whenever suitable.*
☒	☐	For Bayesian analysis, information on the choice of priors and Markov chain Monte Carlo settings
☐	☒	For hierarchical and complex designs, identification of the appropriate level for tests and full reporting of outcomes
☐	☒	Estimates of effect sizes (e.g. Cohen's *d*, Pearson's *r*), indicating how they were calculated
☐	☒	Clearly defined error bars<br>*State explicitly what error bars represent (e.g. SD, SE, CI)*

Our web collection on statistics for biologists may be useful.

Software and code

Policy information about availability of computer code

Data collection	bcl2fastq2 v2.19 conversion software (Illumina) to convert images (BCL) into sequencing reads (FASTQ); SRA toolkit (NCBI) to download external deep sequencing datasets; and LC480II (Roche) software to collect qPCR data.
Data analysis	Deep sequencing data: Picard (Broad Institute) for library metrics and marking redundant reads; trim_galore v0.4.2 (Babraham Institute, UK) for trimming reads; Bowtie v1.0.1, Bowtie2 v2.2.9 and STAR v2.5.3a for mapping reads; VERSE v0.1.5 for counting gene-specific reads; FLASH v1.2.11 for merging overlapping paired-end reads; HOMER v4.8.3 for post-alignment analysis; samtools v1.3.1 and bedtools v2.25.0 for processing alignment and annotation files; RSeQC v2.6.4 and IGV genome browser v2.3.92 for visualisation of deep sequencing data; DESeq2 v1.14.1 and DiffBind v2.4.8 (both in R) for differential expression and DNA binding analysis; GOstats v2.40.0 (in R) and BLAST2GO for gene ontology analysis; and ISMARA v1.2.2 for motif analysis. Genome analysis: RepeatMasker v4.0.6, crossmatch search engine v1.090518, CENSOR v4.2.29 and Tandem Repeat Finder v4.09 for masking genomic repeats. qPCR analysis: Excel 15.39 and Prism7. The following platforms were used for various data analyses and data visualisations: R v3.4.1 / Bioconductor v3.5, Perl v5.18.2 or Python v2.7.12

For manuscripts utilizing custom algorithms or software that are central to the research but not yet described in published literature, software must be made available to editors/reviewers upon request. We strongly encourage code deposition in a community repository (e.g. GitHub). See the Nature Research guidelines for submitting code & software for further information.

Data

Policy information about availability of data

All manuscripts must include a data availability statement. This statement should provide the following information, where applicable:

- Accession codes, unique identifiers, or web links for publicly available datasets
- A list of figures that have associated raw data
- A description of any restrictions on data availability

Raw and processed deep sequencing data will be available from Gene Expression Omnibus (GEO) under accession number GSE113186.

Field-specific reporting

Please select the best fit for your research. If you are not sure, read the appropriate sections before making your selection.

☒ Life sciences ☐ Behavioural & social sciences ☐ Ecological, evolutionary & environmental sciences

For a reference copy of the document with all sections, see nature.com/authors/policies/ReportingSummary-flat.pdf

Life sciences study design

All studies must disclose on these points even when the disclosure is negative.

Sample size	No statistical method was used for determining sample size. We followed the literature to select the appropriate sample size.
Data exclusions	Unviable embryos and tissues were excluded from our analyses. We also excluded trials of establishing and optimising next-generation sequencing protocols (ChIP-Seq, DNase-Seq and next-generation capture-C).
Replication	Once the protocols were established, all attempts of replication were successful.
Randomization	Due to the nature of experiments, there was no need of randomization.
Blinding	Due to the nature of experiments, the authors were not blinded to allocation during experiments and results analysis.

Reporting for specific materials, systems and methods

Materials & experimental systems

n/a	Involved in the study	
☐	☒	Unique biological materials
☐	☒	Antibodies
☒	☐	Eukaryotic cell lines
☒	☐	Palaeontology
☐	☒	Animals and other organisms
☒	☐	Human research participants

Methods

n/a	Involved in the study	
☐	☒	ChIP-seq
☒	☐	Flow cytometry
☒	☐	MRI-based neuroimaging

Unique biological materials

Policy information about availability of materials

Obtaining unique materials	Affinity-purified rabbit polyclonal antibodies were generated against different amino acid sequences of X. tropicalis Tbx6. These antibodies will be available from the authors upon request.

Antibodies

Antibodies used	Rabbit polyclonal anti-beta-catenin (Santa-Cruz, H-102), rabbit polyclonal anti-H3K4me1 (Abcam, ab8895), rabbit polyclonal anti-HA (Abcam, ab9110), mouse monoclonal anti-RNAPII (Covance, MMS-126R), rabbit polyclonal anti-Sox3 (Klymkowsky Lab); rabbit polyclonal anti-VegT (Gurdon Lab), rabbit polyclonal anti-Tbx6 (our lab, #4596 and #5061), mouse monoclonal anti-alfa-Tubulin (Sigma, T5168), rabbit polyclonal anti phospho-Smad1/5/9 (Cell Signalling, #9511), goat polyclonal Smad2/3 (R&D Systems, AF3797), TrueBlot HRP-conjugated anti-rabbit IgG (Rockland Immunochemicals, 18-8816-31), normal goat HRP-conjugated anti-

	mouse IgG (Thermo Fisher Scientific, 31430), goat anti-rabbit IgG-HRP (ThermoFisher Scientific, G-21234), donkey anti-goat IgG-HRP (Santa Cruz Biotechnology, sc-2020).
Validation	All antibodies have been validated for Xenopus.<br>Anti-beta-catenin (H-102) validated in our lab and by Nakamura et al. (2016) for ChIP.<br>Anti-H3K4me1 (ab8895): validated in our lab for ChIP.<br>Anti-HA (ab9110): validated in our lab for ChIP.<br>Anti-RNAPII (MMS-126R): validated in our lab for ChIP.<br>Anti-Sox3 (Klymkowsky Lab): validated in our lab for WMIHC (1:1,000), IP, WB (1:2,000) and ChIP.<br>Anti-VegT (Gurdon Lab): validated in our lab for IP, WB (1:2,000) and ChIP; validated by Stennard et al. (1999) for WMIHC.<br>Anti-Tbx6 (our lab, #4596 and #5061): validated in our lab for IP (#4596), WB (#5061, 1:2,000) and ChIP (#4596).<br>Anti-alfa-Tubulin (T5168): validated in our lab for WB (1:5,000).<br>Anti-phospho-Smad1/5/9 (#9511): validated in our lab for WMIHC (1:500) and ChIP.<br>Anti-Smad2/3 (AF3797): validated in our lab for WMIHC (1:500) and ChIP.

Animals and other organisms

Policy information about studies involving animals; ARRIVE guidelines recommended for reporting animal research

Laboratory animals	Outbred X. tropicalis and X. laevis were obtained from Nasco (Wisconsin, USA)
Wild animals	This study did not involve wild animals.
Field-collected samples	This study did not involve collecting samples from the field.

ChIP-seq

Data deposition

☒ Confirm that both raw and final processed data have been deposited in a public database such as GEO.

☒ Confirm that you have deposited or provided access to graph files (e.g. BED files) for the called peaks.

Data access links<br>*May remain private before publication.*	https://www.ncbi.nlm.nih.gov/geo/query/acc.cgi?acc=GSE113186<br>Reviewer token: yrkjyomkzlctfcr
Files in database submission	HA_st10p_oe_myodHA_rep1.fastq.gz<br>HA_st10p_oe_myodHA_rep2.fastq.gz<br>sox3_st10p_oe_myodHA_rep1.fastq.gz<br>sox3_st10p_oe_myodHA_rep2.fastq.gz<br>pol2_st10p_oe_myodHA_rep1.fastq.gz<br>pol2_st10p_oe_myodHA_rep2.fastq.gz<br>smad2_st8p_ctrl_rep1.fastq.gz<br>smad2_st8p_ctrl_rep2.fastq.gz<br>smad2_st8p_mPSkd_rep1.fastq.gz<br>smad2_st8p_mPSkd_rep2.fastq.gz<br>bcat_st9p_ctrl_rep1.fastq.gz<br>bcat_st9p_ctrl_rep2.fastq.gz<br>bcat_st9p_mPSkd_rep1.fastq.gz<br>bcat_st9p_mPSkd_rep2.fastq.gz<br>pol2_st8p_ctrl_rep1.fastq.gz<br>pol2_st8p_ctrl_rep2.fastq.gz<br>pol2_st8p_mPSkd_rep1.fastq.gz<br>pol2_st8p_mPSkd_rep2.fastq.gz<br>h3k4me1_st8p_ctrl_rep1.fastq.gz<br>h3k4me1_st8p_ctrl_rep2.fastq.gz<br>h3k4me1_st8p_mPSkd_rep1.fastq.gz<br>h3k4me1_st8p_mPSkd_rep2.fastq.gz<br>input_st6.fastq.gz<br>input_st7.fastq.gz<br>input_st8_rep1_r1.fastq.gz<br>input_st8_rep1_r2.fastq.gz<br>input_st8_rep2.fastq.gz<br>input_st8p_rep1.fastq.gz<br>input_st8p_rep2.fastq.gz<br>input_st9p.fastq.gz<br>input_st10p_rep1.fastq.gz<br>input_st10p_rep2.fastq.gz<br>input_st10p_rep3.fastq.gz<br>input_st11.fastq.gz<br>input_st12p_rep1.fastq.gz<br>input_st12p_rep2_r1.fastq.gz<br>input_st12p_rep2_r2.fastq.gz

April 2018

pol2_st6.fastq.gz
pol2_st7.fastq.gz
pol2_st8.fastq.gz
pol2_st8p.fastq.gz
pol2_st11.fastq.gz
pol2_st12p.fastq.gz
pol2_st13p.fastq.gz
bcat_st6.fastq.gz
bcat_st8.fastq.gz
bcat_st10p.fastq.gz
smad1_st8p.fastq.gz
smad1_st10p_rep1.fastq.gz
smad1_st10p_rep2.fastq.gz
smad2_st8.fastq.gz
smad2_st10p.fastq.gz
sox3_st8_r1.fastq.gz
sox3_st8_r2.fastq.gz
sox3_st10p.fastq.gz
sox3_st12p_r1.fastq.gz
sox3_st12p_r2.fastq.gz
sox3_st20_head.fastq.gz
sox3_st20_trunk.fastq.gz
sox3_st20_bud.fastq.gz
vegT_st8.fastq.gz
vegT_st10p.fastq.gz
tbx6_st20.fastq.gz
input_st13p.fastq.gz
input_st20.fastq.gz
input_st20_head.fastq.gz
input_st20_trunk.fastq.gz
input_st20_bud.fastq.gz
dnase_st8p_ctrl_rep1.fastq.gz
dnase_st8p_ctrl_rep2_r1.fastq.gz
dnase_st8p_ctrl_rep2_r2.fastq.gz
dnase_st8p_mPSkd_rep1.fastq.gz
dnase_st8p_mPSkd_rep2_r1.fastq.gz
dnase_st8p_mPSkd_rep2_r2.fastq.gz
dnase_st8p_naked_gDNA_rep1.fastq.gz
dnase_st8p_naked_gDNA_rep2_r1.fastq.gz
dnase_st8p_naked_gDNA_rep2_r2.fastq.gz
capC_st8p_ctrl_rep1_r1.fastq.gz
capC_st8p_ctrl_rep1_r2.fastq.gz
capC_st8p_ctrl_rep2_r1.fastq.gz
capC_st8p_ctrl_rep2_r2.fastq.gz
capC_st8p_ctrl_rep3_r1.fastq.gz
capC_st8p_ctrl_rep3_r2.fastq.gz
capC_st8p_mPSkd_rep1_r1.fastq.gz
capC_st8p_mPSkd_rep1_r2.fastq.gz
capC_st8p_mPSkd_rep2_r1.fastq.gz
capC_st8p_mPSkd_rep2_r2.fastq.gz
capC_st8p_mPSkd_rep3_r1.fastq.gz
capC_st8p_mPSkd_rep3_r2.fastq.gz
amanitin_st8p_frogA_GG198_R1.fastq.gz
amanitin_st8p_frogA_GG198_R2.fastq.gz
amanitin_st8p_frogB_GG199_R1.fastq.gz
amanitin_st8p_frogB_GG199_R2.fastq.gz
amanitin_st8p_frogC_GG200_R1.fastq.gz
amanitin_st8p_frogC_GG200_R2.fastq.gz
amanitin_st9p_frogA_GG204_R1.fastq.gz
amanitin_st9p_frogA_GG204_R2.fastq.gz
amanitin_st9p_frogB_GG205_R1.fastq.gz
amanitin_st9p_frogB_GG205_R2.fastq.gz
amanitin_st9p_frogC_GG206_R1.fastq.gz
amanitin_st9p_frogC_GG206_R2.fastq.gz
amanitin_st10p_frogA_GG210_R1.fastq.gz
amanitin_st10p_frogA_GG210_R2.fastq.gz
amanitin_st10p_frogB_GG211_R1.fastq.gz
amanitin_st10p_frogB_GG211_R2.fastq.gz
amanitin_st10p_frogC_GG212_R1.fastq.gz
amanitin_st10p_frogC_GG212_R2.fastq.gz
uni_st8p_frogA_GG201_R1.fastq.gz
uni_st8p_frogA_GG201_R2.fastq.gz
uni_st8p_frogB_GG202_R1.fastq.gz
uni_st8p_frogB_GG202_R2.fastq.gz
uni_st8p_frogC_GG203_R1.fastq.gz

uni_st8p_frogC_GG203_R2.fastq.gz
uni_st9p_frogA_GG207_R1.fastq.gz
uni_st9p_frogA_GG207_R2.fastq.gz
uni_st9p_frogB_GG208_R1.fastq.gz
uni_st9p_frogB_GG208_R2.fastq.gz
uni_st9p_frogC_GG209_R1.fastq.gz
uni_st9p_frogC_GG209_R2.fastq.gz
uni_st10p_frogA_GG213_R1.fastq.gz
uni_st10p_frogA_GG213_R2.fastq.gz
uni_st10p_frogB_GG214_R1.fastq.gz
uni_st10p_frogB_GG214_R2.fastq.gz
uni_st10p_frogC_GG215_R1.fastq.gz
uni_st10p_frogC_GG215_R2.fastq.gz
WntLOF_st8p_frogA_GG028_R1.fastq.gz
WntLOF_st8p_frogA_GG028_R2.fastq.gz
WntLOF_st8p_frogB_GG029_R1.fastq.gz
WntLOF_st8p_frogB_GG029_R2.fastq.gz
WntLOF_st8p_frogC_GG030_R1.fastq.gz
WntLOF_st8p_frogC_GG030_R2.fastq.gz
WntLOF_st9p_frogA_GG031_R1.fastq.gz
WntLOF_st9p_frogA_GG031_R2.fastq.gz
WntLOF_st9p_frogB_GG032_R1.fastq.gz
WntLOF_st9p_frogB_GG032_R2.fastq.gz
WntLOF_st9p_frogC_GG033_R1.fastq.gz
WntLOF_st9p_frogC_GG033_R2.fastq.gz
WntLOF_st10p_frogA_GG034_R1.fastq.gz
WntLOF_st10p_frogA_GG034_R2.fastq.gz
WntLOF_st10p_frogB_GG035_R1.fastq.gz
WntLOF_st10p_frogB_GG035_R2.fastq.gz
WntLOF_st10p_frogC_GG036_R1.fastq.gz
WntLOF_st10p_frogC_GG036_R2.fastq.gz
cMO_st8p_frogA_GG037_R1.fastq.gz
cMO_st8p_frogA_GG037_R2.fastq.gz
cMO_st8p_frogB_GG038_R1.fastq.gz
cMO_st8p_frogB_GG038_R2.fastq.gz
cMO_st8p_frogC_GG039_R1.fastq.gz
cMO_st8p_frogC_GG039_R2.fastq.gz
cMO_st9p_frogA_GG040_R1.fastq.gz
cMO_st9p_frogA_GG040_R2.fastq.gz
cMO_st9p_frogB_GG041_R1.fastq.gz
cMO_st9p_frogB_GG041_R2.fastq.gz
cMO_st9p_frogC_GG042_R1.fastq.gz
cMO_st9p_frogC_GG042_R2.fastq.gz
cMO_st10p_frogA_GG043_R1.fastq.gz
cMO_st10p_frogA_GG043_R2.fastq.gz
cMO_st10p_frogB_GG044_R1.fastq.gz
cMO_st10p_frogB_GG044_R2.fastq.gz
cMO_st10p_frogC_GG045_R1.fastq.gz
cMO_st10p_frogC_GG045_R2.fastq.gz
uni_st8p_frogA_GG046_R1.fastq.gz
uni_st8p_frogA_GG046_R2.fastq.gz
uni_st8p_frogB_GG047_R1.fastq.gz
uni_st8p_frogB_GG047_R2.fastq.gz
uni_st8p_frogC_GG048_R1.fastq.gz
uni_st8p_frogC_GG048_R2.fastq.gz
uni_st9p_frogA_GG049_R1.fastq.gz
uni_st9p_frogA_GG049_R2.fastq.gz
uni_st9p_frogB_GG050_R1.fastq.gz
uni_st9p_frogB_GG050_R2.fastq.gz
uni_st9p_frogC_GG051_R1.fastq.gz
uni_st9p_frogC_GG051_R2.fastq.gz
uni_st10p_frogA_GG052_R1.fastq.gz
uni_st10p_frogA_GG052_R2.fastq.gz
uni_st10p_frogB_GG053_R1.fastq.gz
uni_st10p_frogB_GG053_R2.fastq.gz
uni_st10p_frogC_GG054_R1.fastq.gz
uni_st10p_frogC_GG054_R2.fastq.gz
NodalLOF_st8p_frogA_GG055_R1.fastq.gz
NodalLOF_st8p_frogA_GG055_R2.fastq.gz
NodalLOF_st8p_frogB_GG056_R1.fastq.gz
NodalLOF_st8p_frogB_GG056_R2.fastq.gz
NodalLOF_st8p_frogC_GG057_R1.fastq.gz
NodalLOF_st8p_frogC_GG057_R2.fastq.gz
NodalLOF_st9p_frogA_GG058_R1.fastq.gz
NodalLOF_st9p_frogA_GG058_R2.fastq.gz

NodalLOF_st9p_frogB_GG059_R1.fastq.gz
NodalLOF_st9p_frogB_GG059_R2.fastq.gz
NodalLOF_st9p_frogC_GG060_R1.fastq.gz
NodalLOF_st9p_frogC_GG060_R2.fastq.gz
NodalLOF_st10p_frogA_GG061_R1.fastq.gz
NodalLOF_st10p_frogA_GG061_R2.fastq.gz
NodalLOF_st10p_frogB_GG062_R1.fastq.gz
NodalLOF_st10p_frogB_GG062_R2.fastq.gz
NodalLOF_st10p_frogC_GG063_R1.fastq.gz
NodalLOF_st10p_frogC_GG063_R2.fastq.gz
BmpLOF_st8p_frogA_GG064_R1.fastq.gz
BmpLOF_st8p_frogA_GG064_R2.fastq.gz
BmpLOF_st8p_frogB_GG065_R1.fastq.gz
BmpLOF_st8p_frogB_GG065_R2.fastq.gz
BmpLOF_st8p_frogC_GG066_R1.fastq.gz
BmpLOF_st8p_frogC_GG066_R2.fastq.gz
BmpLOF_st9p_frogA_GG067_R1.fastq.gz
BmpLOF_st9p_frogA_GG067_R2.fastq.gz
BmpLOF_st9p_frogB_GG068_R1.fastq.gz
BmpLOF_st9p_frogB_GG068_R2.fastq.gz
BmpLOF_st9p_frogC_GG069_R1.fastq.gz
BmpLOF_st9p_frogC_GG069_R2.fastq.gz
BmpLOF_st10p_frogA_GG070_R1.fastq.gz
BmpLOF_st10p_frogA_GG070_R2.fastq.gz
BmpLOF_st10p_frogB_GG071_R1.fastq.gz
BmpLOF_st10p_frogB_GG071_R2.fastq.gz
BmpLOF_st10p_frogC_GG072_R1.fastq.gz
BmpLOF_st10p_frogC_GG072_R2.fastq.gz
dmso_st8p_frogA_GG073_R1.fastq.gz
dmso_st8p_frogA_GG073_R2.fastq.gz
dmso_st8p_frogB_GG074_R1.fastq.gz
dmso_st8p_frogB_GG074_R2.fastq.gz
dmso_st8p_frogC_GG075_R1.fastq.gz
dmso_st8p_frogC_GG075_R2.fastq.gz
dmso_st9p_frogA_GG076_R1.fastq.gz
dmso_st9p_frogA_GG076_R2.fastq.gz
dmso_st9p_frogB_GG077_R1.fastq.gz
dmso_st9p_frogB_GG077_R2.fastq.gz
dmso_st9p_frogC_GG078_R1.fastq.gz
dmso_st9p_frogC_GG078_R2.fastq.gz
dmso_st10p_frogA_GG079_R1.fastq.gz
dmso_st10p_frogA_GG079_R2.fastq.gz
dmso_st10p_frogB_GG080_R1.fastq.gz
dmso_st10p_frogB_GG080_R2.fastq.gz
dmso_st10p_frogC_GG081_R1.fastq.gz
dmso_st10p_frogC_GG081_R2.fastq.gz
mPouVkd_st8p_frogC_GG147_R1.fastq.gz
mPouVkd_st8p_frogC_GG147_R2.fastq.gz
mPouVkd_st8p_frogD_GG148_R1.fastq.gz
mPouVkd_st8p_frogD_GG148_R2.fastq.gz
mPouVkd_st8p_frogE_GG149_R1.fastq.gz
mPouVkd_st8p_frogE_GG149_R2.fastq.gz
mPouVkd_st9p_frogC_GG156_R1.fastq.gz
mPouVkd_st9p_frogC_GG156_R2.fastq.gz
mPouVkd_st9p_frogD_GG157_R1.fastq.gz
mPouVkd_st9p_frogD_GG157_R2.fastq.gz
mPouVkd_st9p_frogE_GG158_R1.fastq.gz
mPouVkd_st9p_frogE_GG158_R2.fastq.gz
mPouVkd_st10p_frogC_GG165_R1.fastq.gz
mPouVkd_st10p_frogC_GG165_R2.fastq.gz
mPouVkd_st10p_frogD_GG166_R1.fastq.gz
mPouVkd_st10p_frogD_GG166_R2.fastq.gz
mPouVkd_st10p_frogE_GG167_R1.fastq.gz
mPouVkd_st10p_frogE_GG167_R2.fastq.gz
mPSkd_st8p_frogC_GG150_R1.fastq.gz
mPSkd_st8p_frogC_GG150_R2.fastq.gz
mPSkd_st8p_frogD_GG151_R1.fastq.gz
mPSkd_st8p_frogD_GG151_R2.fastq.gz
mPSkd_st8p_frogE_GG152_R1.fastq.gz
mPSkd_st8p_frogE_GG152_R2.fastq.gz
mPSkd_st9p_frogC_GG159_R1.fastq.gz
mPSkd_st9p_frogC_GG159_R2.fastq.gz
mPSkd_st9p_frogD_GG160_R1.fastq.gz
mPSkd_st9p_frogD_GG160_R2.fastq.gz
mPSkd_st9p_frogE_GG161_R1.fastq.gz

mPSkd_st9p_frogE_GG161_R2.fastq.gz
mPSkd_st10p_frogC_GG168_R1.fastq.gz
mPSkd_st10p_frogC_GG168_R2.fastq.gz
mPSkd_st10p_frogD_GG169_R1.fastq.gz
mPSkd_st10p_frogD_GG169_R2.fastq.gz
mPSkd_st10p_frogE_GG170_R1.fastq.gz
mPSkd_st10p_frogE_GG170_R2.fastq.gz
uni_st8p_frogC_GG153_R1.fastq.gz
uni_st8p_frogC_GG153_R2.fastq.gz
uni_st8p_frogD_GG154_R1.fastq.gz
uni_st8p_frogD_GG154_R2.fastq.gz
uni_st8p_frogE_GG155_R1.fastq.gz
uni_st8p_frogE_GG155_R2.fastq.gz
uni_st9p_frogC_GG162_R1.fastq.gz
uni_st9p_frogC_GG162_R2.fastq.gz
uni_st9p_frogD_GG163_R1.fastq.gz
uni_st9p_frogD_GG163_R2.fastq.gz
uni_st9p_frogE_GG164_R1.fastq.gz
uni_st9p_frogE_GG164_R2.fastq.gz
uni_st10p_frogC_GG171_R1.fastq.gz
uni_st10p_frogC_GG171_R2.fastq.gz
uni_st10p_frogD_GG172_R1.fastq.gz
uni_st10p_frogD_GG172_R2.fastq.gz
uni_st10p_frogE_GG173_R1.fastq.gz
uni_st10p_frogE_GG173_R2.fastq.gz
mVegTkd_st8p_frogA_TS003_R1.fastq.gz
mVegTkd_st8p_frogA_TS003_R2.fastq.gz
mVegTkd_st8p_frogE_TS012_R1.fastq.gz
mVegTkd_st8p_frogE_TS012_R2.fastq.gz
mVegTkd_st8p_frogF_TS021_R1.fastq.gz
mVegTkd_st8p_frogF_TS021_R2.fastq.gz
mVegTkd_st9p_frogA_TS006_R1.fastq.gz
mVegTkd_st9p_frogA_TS006_R2.fastq.gz
mVegTkd_st9p_frogE_TS015_R1.fastq.gz
mVegTkd_st9p_frogE_TS015_R2.fastq.gz
mVegTkd_st9p_frogF_TS024_R1.fastq.gz
mVegTkd_st9p_frogF_TS024_R2.fastq.gz
mVegTkd_st10p_frogA_TS009_R1.fastq.gz
mVegTkd_st10p_frogA_TS009_R2.fastq.gz
mVegTkd_st10p_frogE_TS018_R1.fastq.gz
mVegTkd_st10p_frogE_TS018_R2.fastq.gz
mVegTkd_st10p_frogF_TS027_R1.fastq.gz
mVegTkd_st10p_frogF_TS027_R2.fastq.gz
cMO_st8p_frogA_TS002_R1.fastq.gz
cMO_st8p_frogA_TS002_R2.fastq.gz
cMO_st8p_frogE_TS011_R1.fastq.gz
cMO_st8p_frogE_TS011_R2.fastq.gz
cMO_st8p_frogF_TS020_R1.fastq.gz
cMO_st8p_frogF_TS020_R2.fastq.gz
cMO_st9p_frogA_TS005_R1.fastq.gz
cMO_st9p_frogA_TS005_R2.fastq.gz
cMO_st9p_frogE_TS014_R1.fastq.gz
cMO_st9p_frogE_TS014_R2.fastq.gz
cMO_st9p_frogF_TS023_R1.fastq.gz
cMO_st9p_frogF_TS023_R2.fastq.gz
cMO_st10p_frogA_TS008_R1.fastq.gz
cMO_st10p_frogA_TS008_R2.fastq.gz
cMO_st10p_frogE_TS017_R1.fastq.gz
cMO_st10p_frogE_TS017_R2.fastq.gz
cMO_st10p_frogF_TS026_R1.fastq.gz
cMO_st10p_frogF_TS026_R2.fastq.gz
uni_st8p_frogA_TS001_R1.fastq.gz
uni_st8p_frogA_TS001_R2.fastq.gz
uni_st8p_frogE_TS010_R1.fastq.gz
uni_st8p_frogE_TS010_R2.fastq.gz
uni_st8p_frogF_TS019_R1.fastq.gz
uni_st8p_frogF_TS019_R2.fastq.gz
uni_st9p_frogA_TS004_R1.fastq.gz
uni_st9p_frogA_TS004_R2.fastq.gz
uni_st9p_frogE_TS013_R1.fastq.gz
uni_st9p_frogE_TS013_R2.fastq.gz
uni_st9p_frogF_TS022_R1.fastq.gz
uni_st9p_frogF_TS022_R2.fastq.gz
uni_st10p_frogA_TS007_R1.fastq.gz
uni_st10p_frogA_TS007_R2.fastq.gz

uni_st10p_frogE_TS016_R1.fastq.gz
uni_st10p_frogE_TS016_R2.fastq.gz
uni_st10p_frogF_TS025_R1.fastq.gz
uni_st10p_frogF_TS025_R2.fastq.gz
pol2_st6_xt7_unique.bw
pol2_st6_xt7.bed
pol2_st7_xt7_unique.bw
pol2_st7_xt7.bed
pol2_st8_xt7_unique.bw
pol2_st8_xt7.bed
pol2_st8p_xt7_unique.bw
pol2_st8p_xt9_unique.bw
pol2_st8p_xt7.bed
pol2_st11_xt7_unique.bw
pol2_st11_xt7.bed
pol2_st12_xt7_unique.bw
pol2_st12_xt7.bed
pol2_st13_xt7_unique.bw
bcat_st6_xt7_unique.bw
bcat_st6_xt7.bed
bcat_st8_xt7_unique.bw
bcat_st8_xt7.bed
bcat_st10p_xt7_unique.bw
bcat_st10p_gentsch_hoppler_xt7_unique.bw
bcat_st10p_xt7.bed
smad1_st8p_xt7_unique.bw
smad1_st8p_xt7.bed
smad1_st10p_xt7_exp12_unique.bw
smad1_st10p_xt7.bed
smad2_st8_xt7_unique.bw
smad2_st8_xt7.bed
smad2_st10p_xt7_unique.bw
smad2_st10p_gentsch_baker_cho_xt7_unique.bw
smad2_st10p_xt7.bed
sox3_st8_xt7_unique.bw
sox3_st8_xt7.bed
sox3_st10p_xt7_unique.bw
sox3_st10p_xt7.bed
sox3_st12p_xt7_unique.bw
sox3_st12p_xt7.bed
sox3_st20_head_xt7_unique.bw
sox3_st20_head_x7.bed
sox3_st20_trunk_xt7_unique.bw
sox3_st20_trunk_x7.bed
sox3_st20_tailbud_xt7_unique.bw
sox3_st20_tailbud_x7.bed
vegT_st8_xt7_unique.bw
vegT_st8_xt7.bed
vegT_st10p_xt7_unique.bw
vegT_st10p_xt7.bed
tbx6_st20_xt7_unique.bw
tbx6_st20_xt7.bed
myod-ha_st10p_oe_myod_xt7_exp12_unique.bw
myod-ha_st10p_oe_myod_xt7.bed
sox3_st10p_oe_myod_xt7_exp12_unique.bw
sox3_st10p_oe_myod_xt7.bed
pol2_st10p_oe_myod_xt7_exp12_unique.bw
pol2_st10p_oe_myod_xt7.bed
smad2_ctrl_st8p_xt7_exp12_unique.bw
smad2_mPSkd_st8p_xt7_exp12_unique.bw
bcat_ctrl_st9p_xt7_exp12_unique.bw
bcat_mPSkd_st9p_xt7_exp12_unique.bw
pol2_ctrl_st8p_xt7_exp12_unique.bw
pol2_mPSkd_st8p_xt7_exp12_unique.bw
h3k4me1_ctrl_st8p_xt7_exp12_unique.bw
h3k4me1_mPSkd_st8p_xt7_exp12_unique.bw
input_xt7_unique.bw
dnase_ctrl_st8p_exp12_xt7_unique.bw
dnase_ctrl_st8p_exp1_xt9_unique.bw
dnase_mPSkd_st8p_exp12_xt7_unique.bw
naked_gDNA_dnase_xt7_unique.bw
dhs_st8p_xt7.bed
capC_ctrl_exp123_xt7.bdg
capC_mPSkd_exp123_xt7.bdg
amanitin_st8p_frogA_GG198.exon.txt

amanitin_st8p_frogA_GG198.intron.txt
amanitin_st8p_frogB_GG199.exon.txt
amanitin_st8p_frogB_GG199.intron.txt
amanitin_st8p_frogC_GG200.exon.txt
amanitin_st8p_frogC_GG200.intron.txt
amanitin_st9p_frogA_GG204.exon.txt
amanitin_st9p_frogA_GG204.intron.txt
amanitin_st9p_frogB_GG205.exon.txt
amanitin_st9p_frogB_GG205.intron.txt
amanitin_st9p_frogC_GG206.exon.txt
amanitin_st9p_frogC_GG206.intron.txt
amanitin_st10p_frogA_GG210.exon.txt
amanitin_st10p_frogA_GG210.intron.txt
amanitin_st10p_frogB_GG211.exon.txt
amanitin_st10p_frogB_GG211.intron.txt
amanitin_st10p_frogC_GG212.exon.txt
amanitin_st10p_frogC_GG212.intron.txt
uni_st8p_frogA_GG201.exon.txt
uni_st8p_frogA_GG201.intron.txt
uni_st8p_frogB_GG202.exon.txt
uni_st8p_frogB_GG202.intron.txt
uni_st8p_frogC_GG203.exon.txt
uni_st8p_frogC_GG203.intron.txt
uni_st9p_frogA_GG207.exon.txt
uni_st9p_frogA_GG207.intron.txt
uni_st9p_frogB_GG208.exon.txt
uni_st9p_frogB_GG208.intron.txt
uni_st9p_frogC_GG209.exon.txt
uni_st9p_frogC_GG209.intron.txt
uni_st10p_frogA_GG213.exon.txt
uni_st10p_frogA_GG213.intron.txt
uni_st10p_frogB_GG214.exon.txt
uni_st10p_frogB_GG214.intron.txt
uni_st10p_frogC_GG215.exon.txt
uni_st10p_frogC_GG215.intron.txt
WntLOF_st8p_frogA_GG028.exon.txt
WntLOF_st8p_frogA_GG028.intron.txt
WntLOF_st8p_frogB_GG029.exon.txt
WntLOF_st8p_frogB_GG029.intron.txt
WntLOF_st8p_frogC_GG030.exon.txt
WntLOF_st8p_frogC_GG030.intron.txt
WntLOF_st9p_frogA_GG031.exon.txt
WntLOF_st9p_frogA_GG031.intron.txt
WntLOF_st9p_frogB_GG032.exon.txt
WntLOF_st9p_frogB_GG032.intron.txt
WntLOF_st9p_frogC_GG033.exon.txt
WntLOF_st9p_frogC_GG033.intron.txt
WntLOF_st10p_frogA_GG034.exon.txt
WntLOF_st10p_frogA_GG034.intron.txt
WntLOF_st10p_frogB_GG035.exon.txt
WntLOF_st10p_frogB_GG035.intron.txt
WntLOF_st10p_frogC_GG036.exon.txt
WntLOF_st10p_frogC_GG036.intron.txt
cMO_st8p_frogA_GG037.exon.txt
cMO_st8p_frogA_GG037.intron.txt
cMO_st8p_frogB_GG038.exon.txt
cMO_st8p_frogB_GG038.intron.txt
cMO_st8p_frogC_GG039.exon.txt
cMO_st8p_frogC_GG039.intron.txt
cMO_st9p_frogA_GG040.exon.txt
cMO_st9p_frogA_GG040.intron.txt
cMO_st9p_frogB_GG041.exon.txt
cMO_st9p_frogB_GG041.intron.txt
cMO_st9p_frogC_GG042.exon.txt
cMO_st9p_frogC_GG042.intron.txt
cMO_st10p_frogA_GG043.exon.txt
cMO_st10p_frogA_GG043.intron.txt
cMO_st10p_frogB_GG044.exon.txt
cMO_st10p_frogB_GG044.intron.txt
cMO_st10p_frogC_GG045.exon.txt
cMO_st10p_frogC_GG045.intron.txt
uni_st8p_frogA_GG046.exon.txt
uni_st8p_frogA_GG046.intron.txt
uni_st8p_frogB_GG047.exon.txt
uni_st8p_frogB_GG047.intron.txt

uni_st8p_frogC_GG048.exon.txt
uni_st8p_frogC_GG048.intron.txt
uni_st9p_frogA_GG049.exon.txt
uni_st9p_frogA_GG049.intron.txt
uni_st9p_frogB_GG050.exon.txt
uni_st9p_frogB_GG050.intron.txt
uni_st9p_frogC_GG051.exon.txt
uni_st9p_frogC_GG051.intron.txt
uni_st10p_frogA_GG052.exon.txt
uni_st10p_frogA_GG052.intron.txt
uni_st10p_frogB_GG053.exon.txt
uni_st10p_frogB_GG053.intron.txt
uni_st10p_frogC_GG054.exon.txt
uni_st10p_frogC_GG054.intron.txt
NodalLOF_st8p_frogA_GG055.exon.txt
NodalLOF_st8p_frogA_GG055.intron.txt
NodalLOF_st8p_frogB_GG056.exon.txt
NodalLOF_st8p_frogB_GG056.intron.txt
NodalLOF_st8p_frogC_GG057.exon.txt
NodalLOF_st8p_frogC_GG057.intron.txt
NodalLOF_st9p_frogA_GG058.exon.txt
NodalLOF_st9p_frogA_GG058.intron.txt
NodalLOF_st9p_frogB_GG059.exon.txt
NodalLOF_st9p_frogB_GG059.intron.txt
NodalLOF_st9p_frogC_GG060.exon.txt
NodalLOF_st9p_frogC_GG060.intron.txt
NodalLOF_st10p_frogA_GG061.exon.txt
NodalLOF_st10p_frogA_GG061.intron.txt
NodalLOF_st10p_frogB_GG062.exon.txt
NodalLOF_st10p_frogB_GG062.intron.txt
NodalLOF_st10p_frogC_GG063.exon.txt
NodalLOF_st10p_frogC_GG063.intron.txt
BmpLOF_st8p_frogA_GG064.exon.txt
BmpLOF_st8p_frogA_GG064.intron.txt
BmpLOF_st8p_frogB_GG065.exon.txt
BmpLOF_st8p_frogB_GG065.intron.txt
BmpLOF_st8p_frogC_GG066.exon.txt
BmpLOF_st8p_frogC_GG066.intron.txt
BmpLOF_st9p_frogA_GG067.exon.txt
BmpLOF_st9p_frogA_GG067.intron.txt
BmpLOF_st9p_frogB_GG068.exon.txt
BmpLOF_st9p_frogB_GG068.intron.txt
BmpLOF_st9p_frogC_GG069.exon.txt
BmpLOF_st9p_frogC_GG069.intron.txt
BmpLOF_st10p_frogA_GG070.exon.txt
BmpLOF_st10p_frogA_GG070.intron.txt
BmpLOF_st10p_frogB_GG071.exon.txt
BmpLOF_st10p_frogB_GG071.intron.txt
BmpLOF_st10p_frogC_GG072.exon.txt
BmpLOF_st10p_frogC_GG072.intron.txt
dmso_st8p_frogA_GG073.exon.txt
dmso_st8p_frogA_GG073.intron.txt
dmso_st8p_frogB_GG074.exon.txt
dmso_st8p_frogB_GG074.intron.txt
dmso_st8p_frogC_GG075.exon.txt
dmso_st8p_frogC_GG075.intron.txt
dmso_st9p_frogA_GG076.exon.txt
dmso_st9p_frogA_GG076.intron.txt
dmso_st9p_frogB_GG077.exon.txt
dmso_st9p_frogB_GG077.intron.txt
dmso_st9p_frogC_GG078.exon.txt
dmso_st9p_frogC_GG078.intron.txt
dmso_st10p_frogA_GG079.exon.txt
dmso_st10p_frogA_GG079.intron.txt
dmso_st10p_frogB_GG080.exon.txt
dmso_st10p_frogB_GG080.intron.txt
dmso_st10p_frogC_GG081.exon.txt
dmso_st10p_frogC_GG081.intron.txt
mPouVkd_st8p_frogC_GG147.exon.txt
mPouVkd_st8p_frogC_GG147.intron.txt
mPouVkd_st8p_frogD_GG148.exon.txt
mPouVkd_st8p_frogD_GG148.intron.txt
mPouVkd_st8p_frogE_GG149.exon.txt
mPouVkd_st8p_frogE_GG149.intron.txt
mPouVkd_st9p_frogC_GG156.exon.txt

mPouVkd_st9p_frogC_GG156.intron.txt
mPouVkd_st9p_frogD_GG157.exon.txt
mPouVkd_st9p_frogD_GG157.intron.txt
mPouVkd_st9p_frogE_GG158.exon.txt
mPouVkd_st9p_frogE_GG158.intron.txt
mPouVkd_st10p_frogC_GG165.exon.txt
mPouVkd_st10p_frogC_GG165.intron.txt
mPouVkd_st10p_frogD_GG166.exon.txt
mPouVkd_st10p_frogD_GG166.intron.txt
mPouVkd_st10p_frogE_GG167.exon.txt
mPouVkd_st10p_frogE_GG167.intron.txt
mPSkd_st8p_frogC_GG150.exon.txt
mPSkd_st8p_frogC_GG150.intron.txt
mPSkd_st8p_frogD_GG151.exon.txt
mPSkd_st8p_frogD_GG151.intron.txt
mPSkd_st8p_frogE_GG152.exon.txt
mPSkd_st8p_frogE_GG152.intron.txt
mPSkd_st9p_frogC_GG159.exon.txt
mPSkd_st9p_frogC_GG159.intron.txt
mPSkd_st9p_frogD_GG160.exon.txt
mPSkd_st9p_frogD_GG160.intron.txt
mPSkd_st9p_frogE_GG161.exon.txt
mPSkd_st9p_frogE_GG161.intron.txt
mPSkd_st10p_frogC_GG168.exon.txt
mPSkd_st10p_frogC_GG168.intron.txt
mPSkd_st10p_frogD_GG169.exon.txt
mPSkd_st10p_frogD_GG169.intron.txt
mPSkd_st10p_frogE_GG170.exon.txt
mPSkd_st10p_frogE_GG170.intron.txt
uni_st8p_frogC_GG153.exon.txt
uni_st8p_frogC_GG153.intron.txt
uni_st8p_frogD_GG154.exon.txt
uni_st8p_frogD_GG154.intron.txt
uni_st8p_frogE_GG155.exon.txt
uni_st8p_frogE_GG155.intron.txt
uni_st9p_frogC_GG162.exon.txt
uni_st9p_frogC_GG162.intron.txt
uni_st9p_frogD_GG163.exon.txt
uni_st9p_frogD_GG163.intron.txt
uni_st9p_frogE_GG164.exon.txt
uni_st9p_frogE_GG164.intron.txt
uni_st10p_frogC_GG171.exon.txt
uni_st10p_frogC_GG171.intron.txt
uni_st10p_frogD_GG172.exon.txt
uni_st10p_frogD_GG172.intron.txt
uni_st10p_frogE_GG173.exon.txt
uni_st10p_frogE_GG173.intron.txt
mVegTkd_st8p_frogA_TS003.exon.txt
mVegTkd_st8p_frogA_TS003.intron.txt
mVegTkd_st8p_frogE_TS012.exon.txt
mVegTkd_st8p_frogE_TS012.intron.txt
mVegTkd_st8p_frogF_TS021.exon.txt
mVegTkd_st8p_frogF_TS021.intron.txt
mVegTkd_st9p_frogA_TS006.exon.txt
mVegTkd_st9p_frogA_TS006.intron.txt
mVegTkd_st9p_frogE_TS015.exon.txt
mVegTkd_st9p_frogE_TS015.intron.txt
mVegTkd_st9p_frogF_TS024.exon.txt
mVegTkd_st9p_frogF_TS024.intron.txt
mVegTkd_st10p_frogA_TS009.exon.txt
mVegTkd_st10p_frogA_TS009.intron.txt
mVegTkd_st10p_frogE_TS018.exon.txt
mVegTkd_st10p_frogE_TS018.intron.txt
mVegTkd_st10p_frogF_TS027.exon.txt
mVegTkd_st10p_frogF_TS027.intron.txt
cMO_st8p_frogA_TS002.exon.txt
cMO_st8p_frogA_TS002.intron.txt
cMO_st8p_frogE_TS011.exon.txt
cMO_st8p_frogE_TS011.intron.txt
cMO_st8p_frogF_TS020.exon.txt
cMO_st8p_frogF_TS020.intron.txt
cMO_st9p_frogA_TS005.exon.txt
cMO_st9p_frogA_TS005.intron.txt
cMO_st9p_frogE_TS014.exon.txt
cMO_st9p_frogE_TS014.intron.txt

cMO_st9p_frogF_TS023.exon.txt
cMO_st9p_frogF_TS023.intron.txt
cMO_st10p_frogA_TS008.exon.txt
cMO_st10p_frogA_TS008.intron.txt
cMO_st10p_frogE_TS017.exon.txt
cMO_st10p_frogE_TS017.intron.txt
cMO_st10p_frogF_TS026.exon.txt
cMO_st10p_frogF_TS026.intron.txt
uni_st8p_frogA_TS001.exon.txt
uni_st8p_frogA_TS001.intron.txt
uni_st8p_frogE_TS010.exon.txt
uni_st8p_frogE_TS010.intron.txt
uni_st8p_frogF_TS019.exon.txt
uni_st8p_frogF_TS019.intron.txt
uni_st9p_frogA_TS004.exon.txt
uni_st9p_frogA_TS004.intron.txt
uni_st9p_frogE_TS013.exon.txt
uni_st9p_frogE_TS013.intron.txt
uni_st9p_frogF_TS022.exon.txt
uni_st9p_frogF_TS022.intron.txt
uni_st10p_frogA_TS007.exon.txt
uni_st10p_frogA_TS007.intron.txt
uni_st10p_frogE_TS016.exon.txt
uni_st10p_frogE_TS016.intron.txt
uni_st10p_frogF_TS025.exon.txt
uni_st10p_frogF_TS025.intron.txt

Genome browser session (e.g. UCSC)

We have uploaded genomic tracks (bigWig or BedGraph) to GEO which can be viewed on the UCSC or IGV genome browser. Viewing requires genome assembly 7.1 of X. tropicalis to be installed.

Methodology

Replicates

Specified in Supplementary Table 1. All comparisons between two conditions were carried in biological duplicates or triplicates.

Sequencing depth

Specified in Supplementary Table 1 (overall total: 8.5 billion reads).
ChIP-Seq: 37.5 +/- 16.2 million reads (mean +/- SD), 2.4 billion reads (total).
DNase-Seq: 193.1 +/- 230.9 million reads (mean +/- SD), 1.1 billion reads (total).
Next-generation capture-C: 12.4 +/- 4.5 million reads (mean +/- SD), 74.5 million reads (total).
RNA-Seq: 38.5 +/- 15.5 million reads (mean +/- SD), 4.8 billion reads (total).

Antibodies

Rabbit polyclonal anti-beta-catenin (Santa-Cruz, H-102), rabbit polyclonal anti-H3K4me1 (Abcam, ab8895), rabbit polyclonal anti-HA (Abcam, ab9110), mouse monoclonal anti-RNAPII (Covance, MMS-126R), rabbit polyclonal anti-Sox3 (Klymkowsky Lab); rabbit polyclonal anti-VegT (Gurdon Lab), rabbit polyclonal anti-Tbx6 (our lab, #4596), rabbit polyclonal anti phospho-Smad1/5/9 (Cell Signalling, #9511), and goat polyclonal Smad2/3 (R&D Systems, AF3797).

Peak calling parameters

ChIP-Seq peaks called by HOMER v4.8.3: findpeaks -style factor -minDist 175 -fragLength 175 -inputFragLength 175 -fdr 0.001 -gsize 1.435e9 -F 3 -L 1 -C 0.97. This means that both ChIP and input alignments were extended 3’ to 175 bp for the detection of significant (0.1% FDR) peaks being separated by ≥175 bp. The effective size of the X. tropicalis genome assembly v7.1 was set to 1.435 billion bp, an estimate obtained from the mappability profile. These peaks showed equal or higher tag density than the surrounding 10 kb, ≥3-fold more tags than the input and ≥0.97 unique tag positions relative to the expected number of tags. To detect focal RNAPII recruitment to putative CRMs and avoid calling peaks within broad regions of RNAPII reflecting transcript elongation, the threshold of focal ratio and local enrichment within 10 kb was elevated to 0.6 and 3 (-L 3), respectively. To further eliminate any false positive peaks, we removed any peaks with <0.5 (TFs including signal mediators) or <1 (RNAPII) CPM and those falling into blacklisted regions showing equivocal mappability due to genome assembly errors, gaps or simple/tandem repeats. Regions of equivocal mappability were identified by a two-fold lower (poor) or three-fold higher (excessive) read coverage than the average detected in 400-bp windows sliding at 200-bp intervals through normalised ChIP input and DNase-digested naked genomic DNA.
DNase-Seq peaks called by HOMER v4.8.3: findpeaks -style factor -minDist 100 -fragLength 100 -inputFragLength 100 -fdr 0.001 -gsize 1.435e9 -F 3 -L 1 -C 0.97. This means that alignments of DNase-digested chromatin fragments (or naked genomic DNA fragments considered here as ‘input’) were extended 3’ to 100 bp from the DNase cleavage site to detect significant (0.1% FDR) DNase-mediated read enrichments (hereafter called peaks) being separated by ≥100 bp. The effective size of the X. tropicalis genome assembly v7.1 was set to 1.435 billion bp, an estimate obtained from the mappability of ChIP input reads. These peaks showed equal or higher tag density than the surrounding 10 kb, at least three-fold more tags than the input and ≥0.97 unique tag positions relative to the expected number of tags. Peaks falling into blacklisted regions (see Post-sequencing analysis of ChIP-Seq) were removed.

Data quality

All ChIP-Seq libraries were of high complexity yielding >75% unique reads and low adapter contamination (<5%). in most cases peak calling (0.1% FDR) revealed between several thousands to hundreds of thousands of binding events. De novo motif analysis of peaks revealed the expected DNA recognition sequences of the transcription factor.

Software

HOMER v4.8.3
